# Fe24Ni15Cr3Al-Based AFA Steels in Oxygen-Controlled Liquid Pb at 500 °C, 600 °C, and 700 °C

**DOI:** 10.3390/ma19183826

**Published:** 2026-09-08

**Authors:** Renate Fetzer, Annette Heinzel, Alfons Weisenburger, Georg Müller

**Affiliations:** Institute for Pulsed Power and Microwave Technology (IHM), Karlsruhe Institute of Technology (KIT), Hermann-von-Helmholtz-Platz 1, 76344 Eggenstein-Leopoldshafen, Germany; annette.heinzel@kit.edu (A.H.); alfons.weisenburger@kit.edu (A.W.); georg.mueller@kit.edu (G.M.)

**Keywords:** corrosion, liquid lead, alumina-forming austenitic (AFA) steel, oxidation, dissolution

## Abstract

**Highlights:**

- The investigated AFA steels show oxide scale formation in Pb with 1 × 10^−7^ wt.% dissolved oxygen at up to 600 °C.
- The oxide scale characteristics depend on the surface finish (fine vs coarse ground).
- Dissolution corrosion and Pb penetration are observed after exposure at 700 °C.
- Material differences result in small variations in the oxidation/corrosion behavior.

**Abstract:**

Heavy liquid metals such as liquid lead (Pb) are attractive heat transfer media for advanced energy applications, despite their corrosive nature. In the search for heat-resistant austenitic materials that are corrosion resistant to liquid Pb at high temperature, three new Fe24Ni15Cr3Al-based alumina-forming austenitic (AFA) steels with slightly varying compositions have been developed. The present study investigates the corrosion behavior of these AFA materials using static exposure tests to molten Pb containing 1 × 10^−7^ wt.% dissolved oxygen. The corrosion tests are performed at 500 °C, 600 °C, and 700 °C for 1000 h, 2000 h, and 5000 h each. In addition to the variation in material, temperature, and exposure time, two different surface finishes are also used for the tests. Examination of the specimens after exposure shows the formation of oxide scales for temperatures up to 600 °C on all three AFA materials, with minor material-specific variations. Furthermore, the scale characteristics depend on the surface finish. Coarse ground surfaces exhibit thin Al-rich protective oxide scales, while fine ground surfaces show the formation of thick multilayer oxide scales susceptible to Ni dissolution and Pb penetration. Pb exposure at 700 °C leads to a severe corrosion attack of all three Fe24Ni15Cr3Al-based AFA steels.

## 1. Introduction

Heavy liquid metals such as lead (Pb) or lead-bismuth eutectic (LBE) are attractive heat transfer fluids for advanced energy applications due to their excellent thermal properties and temperature stability over a wide range. Examples are concentrating solar power [1,2,3,4], high-temperature thermal energy storage [5,6], or next-generation (GenIV) nuclear reactors [7]. In the latter case, Pb/LBE offer additional advantages due to their high neutron yield (lead-cooled fast reactor, LRF). One of the main technological challenges to be solved when using liquid Pb or LBE as heat transfer fluid is their corrosivity towards structural materials and related material compatibility issues. Liquid lead (and bismuth) exhibits a high solubility for steel alloying elements, which results in corrosion damage characterized by dissolution and penetration of the liquid metal. With an appropriate management of the amount of oxygen dissolved in the liquid metal, austenitic stainless steels are able to form protective oxide scales at temperatures below 500 °C, while at higher temperatures dissolution corrosion of common steels cannot be avoided by oxygen control only [8,9,10,11,12]. Here, stronger oxide formers such as Al are required to enable the formation of thin, slowly growing protective oxide scales, ideally composed of alumina.

A material family heavily researched in recent years/decades are alumina-forming austenitic (AFA) steels [13,14]. These are heat-resistant austenitic steels that form protective alumina scales instead of chromia when exposed to humid gaseous atmospheres at high temperature (typically > 600 °C). This requires a high enough Al content (supported by the presence of Cr and the third element effect), while the ferrite-stabilizing properties of Al and Cr are counterbalanced by austenite formers (mainly Ni, but also Mn or Cu) to keep the austenitic matrix. Austenitic materials are of particular interest for the development of LFRs because of their enhanced resistance against liquid metal embrittlement compared with ferritic/martensitic steels [15].

AFA steels may be classified according to their Ni content as low-Ni AFA with around 12–15 wt.% Ni (typically Mn and/or Cu are added in this case to stabilize the austenitic phase), “standard” AFA with ca. 20–25 wt.% Ni (carbides are often added for strength), and high-Ni AFA with 30–35 wt.% Ni (characterized by substantial NiAl formation). Due to the high solubility of Ni in liquid Pb/LBE, high-Ni AFA materials with more than 30 wt.% Ni are most likely not suitable for application in heavy liquid metal environments.

Regarding the corrosion behavior of AFA steels with lower Ni content in liquid Pb/LBE, formation of a thin protective oxide scale was reported for an Fe-14Ni-14Cr-2.5Al-2.5Mo AFA steel exposed to Pb (1 × 10^−7^ wt.% dissolved oxygen) at 550 °C, whereas the same material with 20 wt.% Ni showed a slightly inferior performance with localized Ni dissolution [16]. The superior corrosion resistance of alloys with less Ni was confirmed for an Fe-12Ni-15Cr-2.5Al AFA compared with an Fe-20Ni-14Cr-3Al AFA exposed to LBE (1 × 10^−6^ wt.% O, temporarily 1 × 10^−9^ wt.%) at 500 °C [17] and for an Fe-14Ni-14Cr-2.5Al AFA without Mo tested in flowing LBE (1 × 10^−6^ wt.% O) at 550 °C [18]. However, the 14Ni-AFA with Mo partly converted to ferrite (17%) during exposure [16], while the austenite phase of the 12Ni-AFA was stabilized by rather large concentrations of Mn (5 wt.%) and Cu (3 wt.%) [17]. Similarly, an Fe-18Ni-16Cr-4Al duplex steel (78% austenite, 22% ferrite) showed excellent corrosion resistance thanks to formation of a thin protective scale when exposed to LBE (1 × 10^−6^ wt.% O) at 550 °C [19] and to liquid Pb (1 × 10^−6^ wt.% O) at 700 °C [20]. In the latter case, the alloy exhibited an even higher amount of ferrite (25%). 18Ni-AFA steels with a reduced Cr content of 12 wt.% and 2.9 wt.% Al showed formation of a thin protective scale but also accelerated oxidation in liquid Pb (2 × 10^−6^ wt.% O) at 700 °C [21]. A positive effect of Nb and C on the oxide scale formation was reported. Thanks to the reduced Cr content, a stable austenitic matrix was achieved, with inter- and intragranular precipitations (B2-NiAl, Mo-rich Laves phase, NbC) formed after 1000 h of exposure at 700 °C in air [22].

A strategy to keep the austenitic phase during long-term exposure is to use a higher Ni content. Fe-Ni-Cr-Al model alloys with 20–29 wt.% Ni were investigated in Pb (1 × 10^−6^ wt.% O) at 550 °C and 600 °C and showed that for an Al concentration of 2.3–4.3 wt.%, a Cr content of at least 15 wt.% is required to form a thin protective scale [23]. For the alloys with 23 and 28 wt.% Ni, a positive influence of Nb and Y on the oxide scale formation and corrosion resistance was demonstrated in the temperature range 600–650 °C, whereupon superior performance was obtained for 23 wt.% Ni compared with 28 wt.% Ni [24]. Finally, an extensive work was performed on the corrosion behavior of Fe-(23-24)Ni-15Cr-(3-4)Al AFA steels, exploring the influence of dissolved oxygen concentration [25], Al content [26], manufacturing process [27], and exposure temperature [28] on the oxide scale formation in liquid LBE. At the default conditions (1 × 10^−6^ wt.% O, 600 °C), a protective oxide scale composed of an outer Cr-Al-rich layer and an inner Al-rich layer was formed, whereas thicker scales with layers of Fe- and Fe-Cr-based oxides were formed at lower temperature (550 °C) or higher oxygen concentration (1 × 10^−3^ wt.%). The oxide scale formed at 650 °C was not protective, giving rise to dissolution corrosion in some regions. Cold working was found to be beneficial for the oxide scale formation and stability thanks to an enhanced Al diffusion in the bulk material.

Based on the experience from Refs. [23,24], three new AFA materials have been designed within the EU Project INNUMAT [29] by thermodynamic simulations applying the software Thermo-Calc (version 2023a). Since these AFA steels were developed specifically for application in nuclear environments, the use of Co was excluded. The design goals were to obtain phase stable austenitic steels that can be employed at temperatures above 600 °C and to minimize the formation of sigma and other embrittling phases at temperatures above 650 °C. Small additions of V or W were added to contribute to the formation of precipitates that can improve the mechanical properties at high temperature. The amount of Al was kept as low as possible (still high enough to provide corrosion protection) to prevent the long-term formation of ferritic phases. From these studies, three Fe-24Ni-15Cr-3Al-based AFA steels with slightly varying compositions have been developed. In the present study, these three materials are exposed to liquid Pb (1 × 10^−7^ wt.% O) at three different temperatures (500 °C, 600 °C, 700 °C) for up to 5000 h to investigate their corrosion resistance. In addition to the influence of exposure time, temperature, and material composition on the corrosion behavior, the importance of the surface finish is also evaluated.

## 2. Materials and Methods

### 2.1. Materials and Sample Preparation

In this work, three AFA materials were exposed to liquid Pb to investigate their corrosion behavior. The compositions of the tested Fe-24Ni-15Cr-3Al-based AFA steels are given in Table 1. The three alloys (~1.2 kg each) were manufactured at Swerim (Luleå, Sweden) and hot rolled for delivery.

**Figure 1:**
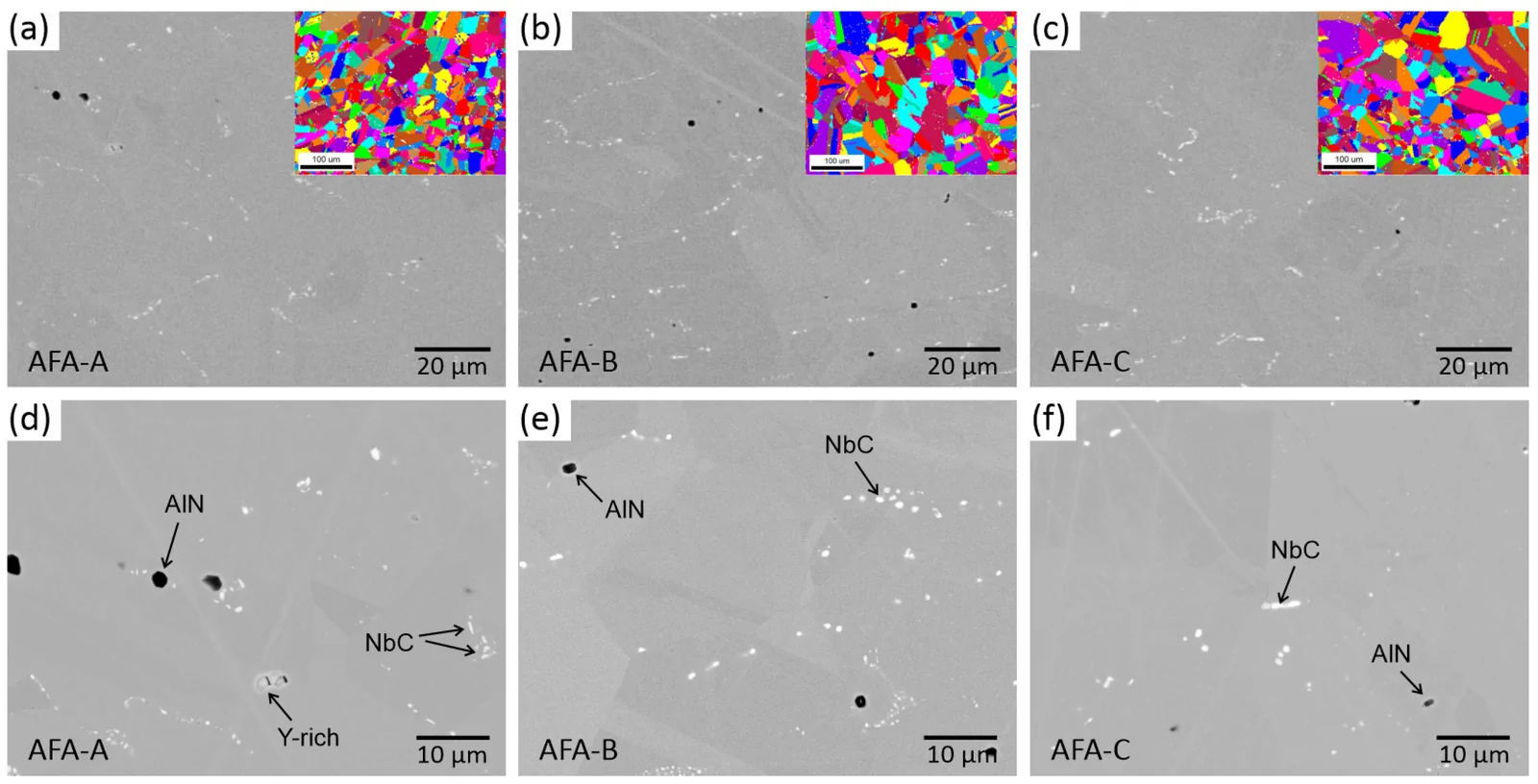
Bulk microstructure of AFA materials prior to exposure. (**a**–**c**) Overview SEM-BSE images (inset: EBSD images) of AFA-A (**a**), AFA-B (**b**), and AFA-C (**c**), (**d**–**f**) SEM-BSE images with higher magnification of AFA-A (**d**), AFA-B (**e**), and AFA-C (**f**).

The as-received AFA steels were then heat-treated at 1150 °C for 30 min and subsequently quenched in air. Cross-section SEM images of the heat-treated AFA materials are shown in Figure 1. All three AFA steels are composed of austenitic grains (see EBSD images for grain size distribution), Nb carbides (NbC), Al nitrides (AlN), and Y-rich inclusions/precipitates. The contamination with N was not intended.

The heat-treated AFA steels were cut into rectangular specimens 25 mm × 11 mm × 2 mm in size (AFA-C: 23 mm × 9 mm × 2 mm) and a hole was drilled in each specimen for mounting during the corrosion test. Then, one side of each specimen was ground with #500 SiC paper (“coarse ground”) and the other side was additionally ground with #2000 SiC paper (“fine ground”). Finally, the specimens were cleaned with ethanol and sonicated in acetone. The RMS surface roughness of the samples prior to exposure was (0.20 ± 0.03) µm on the coarse ground side and (0.050 ± 0.002) µm on the fine ground side as measured on 500 × 300 µm areas with a resolution of 1 pixel/µm using a white light profilometer.

### 2.2. Corrosion Tests and Sample Examination

The exposure tests in molten Pb with a controlled oxygen content of 1 × 10^−7^ wt.% were performed in the COSTA facility (developed and built at Karlsruhe Institute of Technology, Karlsruhe, Germany) [8] for 1000 h, 2000 h, and 5000 h at three different temperatures (500 °C, 600 °C, and 700 °C). Alumina crucibles were filled with lead (Pb, purity 99.99%) and preconditioned to the desired exposure temperature and oxygen content. Hereby, the oxygen control was achieved via the gas phase, where a continuous flow of an Ar/Ar5%H_2_ gas mixture with a defined H_2_/H_2_O partial pressure ratio (0.27 for 500 °C, 1.9 for 600 °C, 10.7 for 700 °C) set the oxygen activity and allowed oxygen removal and supply [12]. After reaching an equilibrium between the oxygen partial pressure in the gas and the dissolved oxygen in the liquid Pb, the specimens were introduced, one per crucible, with the ratio of the exposed specimen surface to the liquid Pb volume below 270 cm^2^/L for all samples. After reaching the end of the test, the samples were extracted from the COSTA facility and allowed to cool to ambient temperature in the controlled atmosphere of a glovebox.

For post-exposure analysis, the specimens were cut into two parts. One part was cold-mounted in resin for cross-sectional analysis. Metallographic preparation of the cross-sections included grinding with SiC paper up to 2400 grit and polishing with a diamond suspension to a final particle size of 1 µm. Selected samples were further polished using a vibration polisher and a 0.25 µm diamond suspension. The prepared cross-sections were examined using a Scanning Electron Microscope (SEM, Zeiss LEO 1530 VP, Oberkochen, Germany) in SE (secondary electrons) and BSE (backscattered electrons) mode with an Energy Dispersive X-ray (EDX) analysis and an Electron Backscatter Diffraction (EBSD) system attached.

The second part of each sample was cleaned of adherent lead and its surface was analyzed by SEM and by X-ray Diffraction (XRD, Seifert 3003 PTS, Rich. Seifert & Co., Ahrensburg, Germany).

## 3. Results

### 3.1. Bulk Evolution

**Figure 2:**
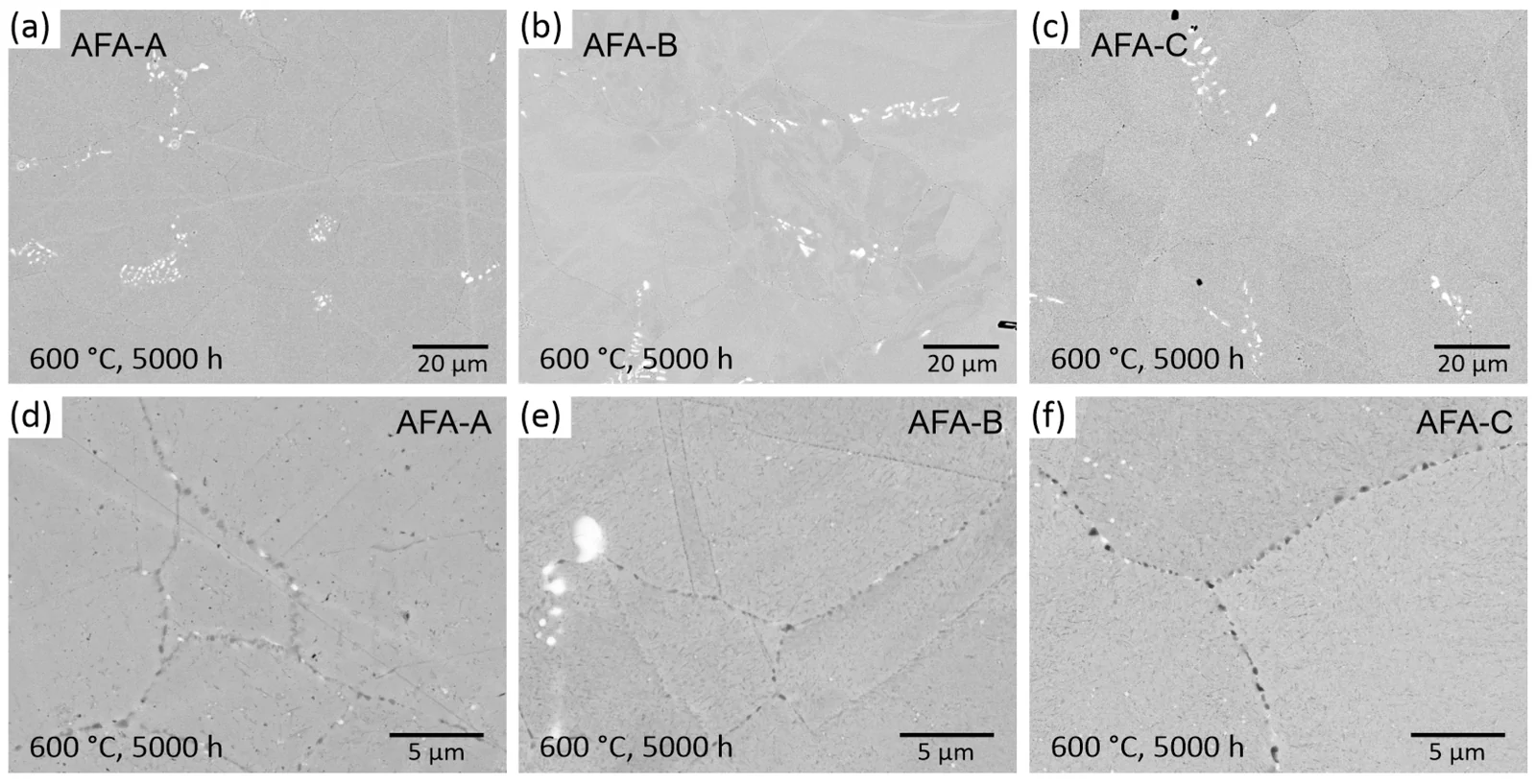
Bulk microstructure of AFA materials after 5000 h exposure at 600 °C. (**a**–**c**) Overview SEM-BSE images of AFA-A (**a**), AFA-B (**b**), and AFA-C (**c**), (**d**–**f**) SEM-BSE images with higher magnification of AFA-A (**d**), AFA-B (**e**), and AFA-C (**f**).

**Figure 3:**
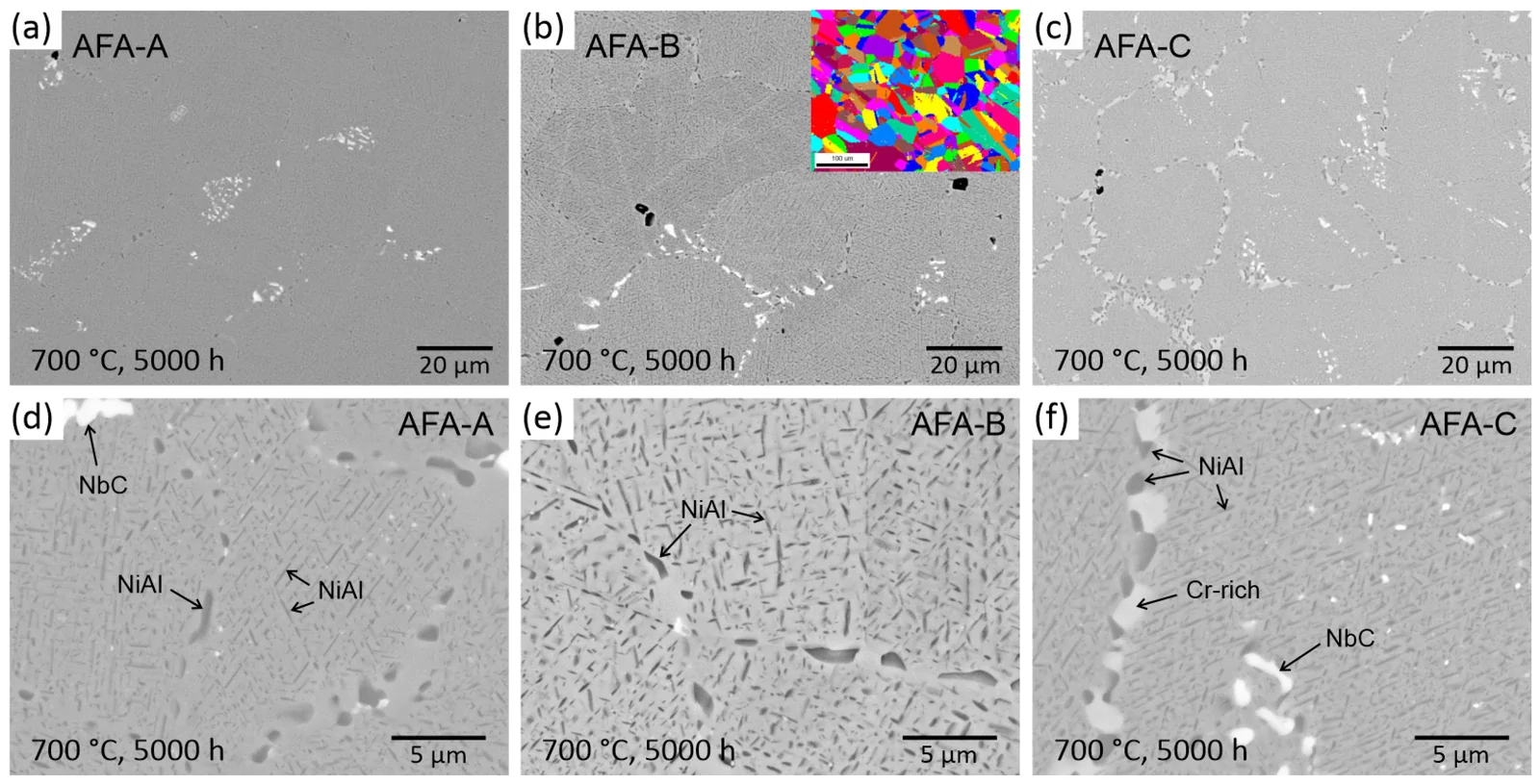
Bulk microstructure of AFA materials after 5000 h exposure at 700 °C. (**a**–**c**) Overview SEM-BSE images (inset: EBSD image) of AFA-A (**a**), AFA-B (**b**), and AFA-C (**c**), (**d**–**f**) SEM-BSE images with higher magnification of AFA-A (**d**), AFA-B (**e**), and AFA-C (**f**).

All three AFA materials keep their austenitic phase upon exposure at 500 °C, 600 °C, and 700 °C. The Nb carbides of all three AFA materials experience coarsening at all temperatures, see the respective images of the bulk after exposure to liquid Pb at 600 °C (Figure 2a–c) and 700 °C (Figure 3a–c) compared with the bulk prior to Pb exposure (Figure 1a–c). However, the grain size distribution does not change significantly, see the results for AFA-B exposed at 700 °C as an example (Figure 3b).

At 600 °C, a slight enrichment of Cr (possibly M_23_C_6_-type carbides) and Al (NiAl) is observed along the grain boundaries of all three AFA materials, see Figure 2d–f. Such an enrichment of specific elements along the grain boundaries is not observed for any exposure at 500 °C.

When exposed at 700 °C, the grain boundaries of all three AFA materials become decorated with many Al-rich precipitates (NiAl, dark), see Figure 3d–f. AFA-C additionally shows rather large Cr-rich areas along the grain boundaries (sigma phase, bright), cf. Figure 3f, which might be related with the presence of W (1 wt.%) in AFA-C. In addition to the changes along the grain boundaries, an elongated secondary NiAl phase is formed within the grains of all three AFA materials (Figure 3d–f).

Intergranular NiAl and Cr_23_C_6_ precipitation has been described previously for AFA steels with similar composition exposed to 600 °C [26]. The formation of intragranular elongated NiAl precipitates during exposure to 650–700 °C is also a commonly observed feature of austenitic steels with similar composition [20,24,30].

### 3.2. Corrosion at 500 °C

#### 3.2.1. Fine Ground Surfaces

**Figure 4:**
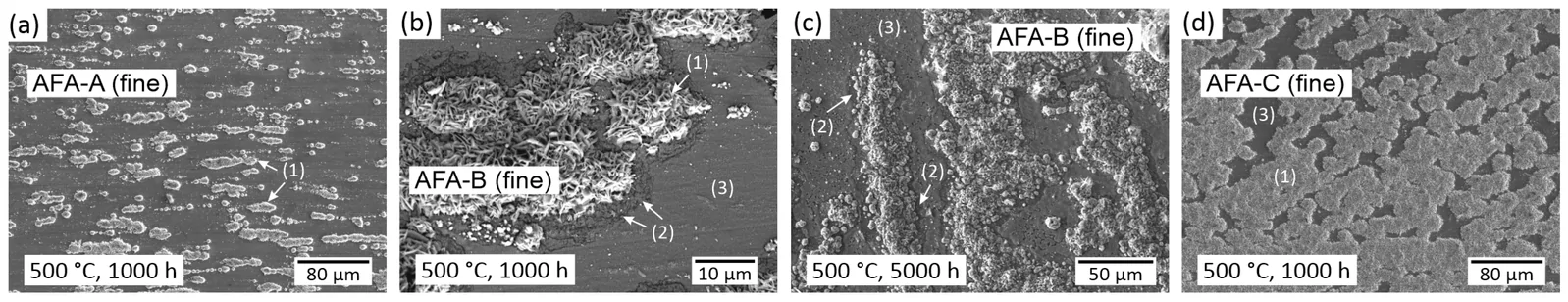
Fine ground surfaces of AFA-A (**a**), AFA-B (**b**,**c**), and AFA-C (**d**) after exposure to liquid Pb at 500 °C, imaged in surface view by SEM-SE. Different corrosion features are observed: (1) oxide nodules, (2) darker region surrounding nodules, (3) unaffected-looking flat region. Further images for other exposure times can be found in the Supplementary Material, Figures S1–S3.

**Figure 5:**
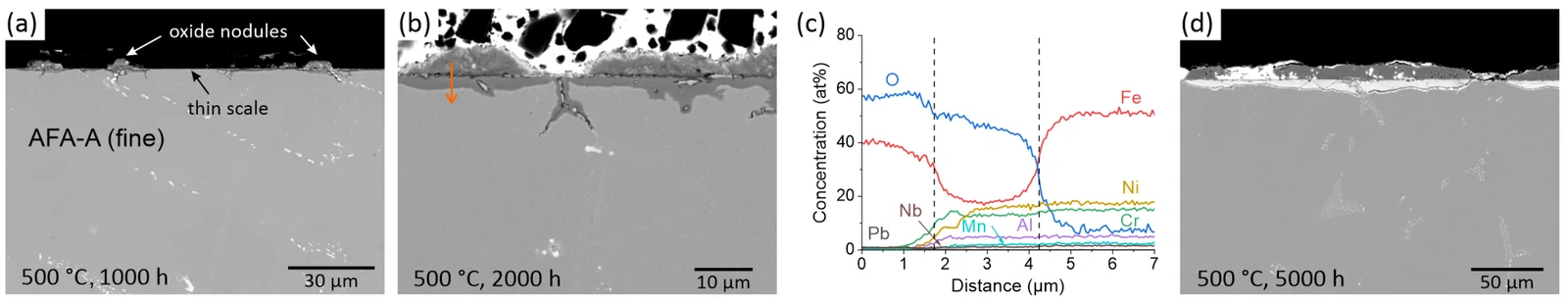
Cross-section analysis of fine ground surface of AFA-A after exposure to liquid Pb at 500 °C. SEM-BSE images after 1000 h exposure (**a**), after 2000 h exposure (orange arrow shows location of line scan) (**b**) with corresponding result of EDX line scan (**c**), and after 5000 h exposure (**d**).

**Figure 6:**
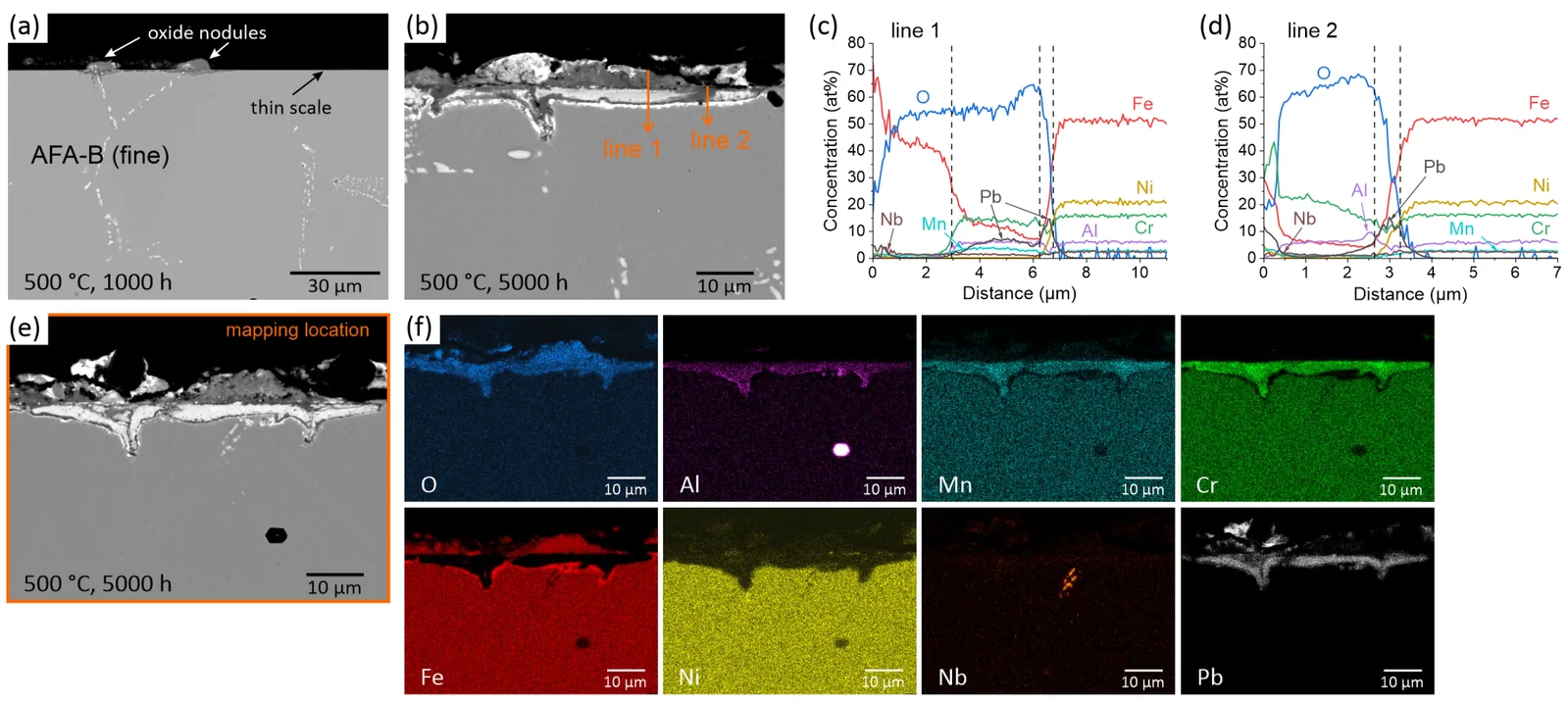
Cross-section analysis of fine ground surface of AFA-B after exposure to liquid Pb at 500 °C. SEM-BSE images after 1000 h (**a**) and after 5000 h exposure (**b**,**e**). EDX elemental compositions along lines shown in (**b**) are presented in (**c**,**d**), EDX elemental mapping result of (**e**) is shown in (**f**).

**Figure 7:**
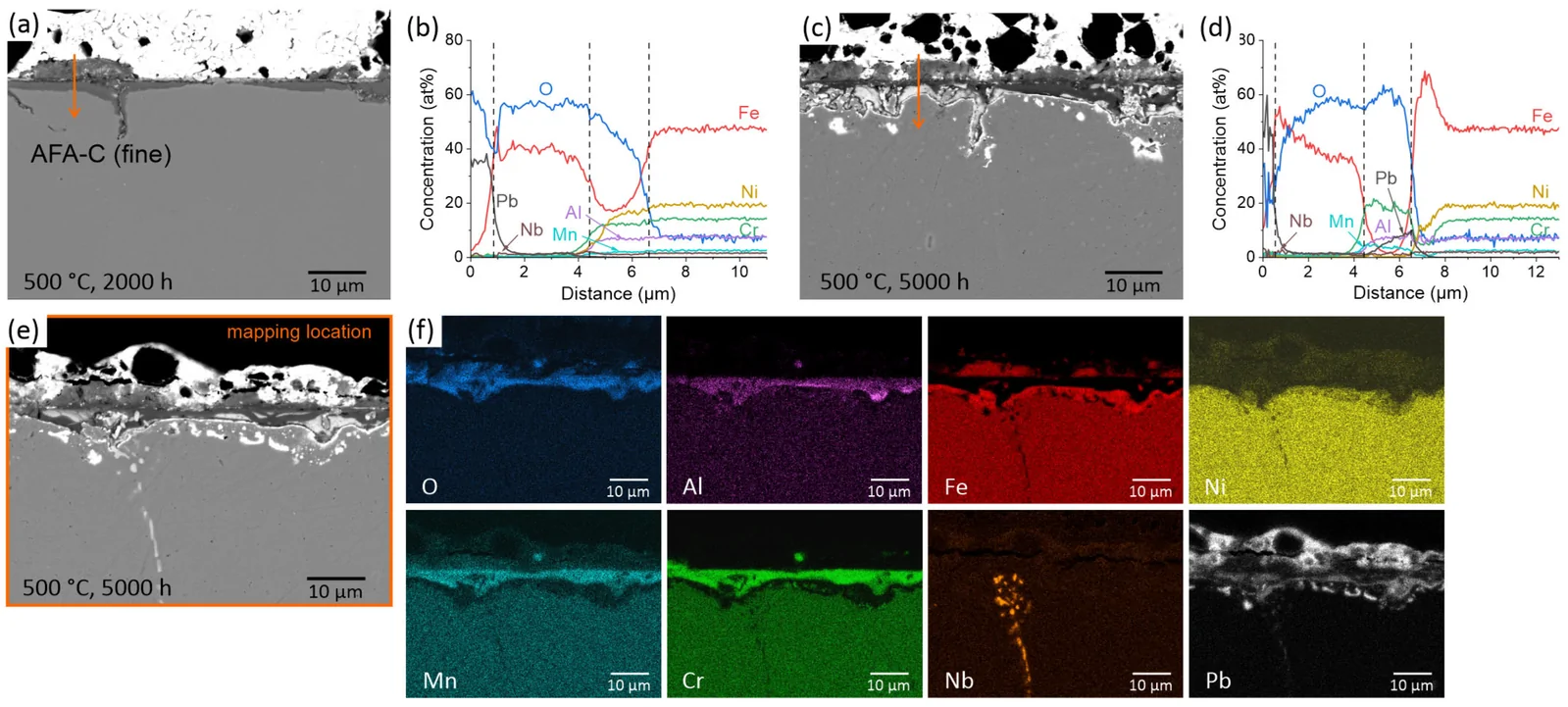
Cross-section analysis of fine ground surface of AFA-C after exposure to liquid Pb at 500 °C. SEM-BSE images after 2000 h exposure (**a**) with corresponding result of EDX line scan (**b**), and after 5000 h exposure (**c**,**e**) with elemental composition along line shown in (**c**) presented in (**d**) and with result of EDX elemental mapping of (**e**) shown in (**f**).

The corrosion behavior of the fine ground surfaces of AFA-A, AFA-B, and AFA-C in liquid Pb at 500 °C is presented in Figure 4 in surface view and in Figure 5, Figure 6 and Figure 7 in cross-section view. Further surface view images can be found in the Supplementary Material in Figures S1–S3.

As apparent from the surface view in Figure 4 and Figures S1–S3, the fine ground surfaces of all three AFA materials are decorated with oxide crystals, which are arranged in hillocks or clusters on top of the original sample surfaces. EDX analysis reveals that the crystal platelets consist of Fe oxide. The number, size, and distribution of the outward-growing Fe oxide nodules across the sample surfaces depend on the material and on the exposure duration. After 1000 h of exposure, AFA-C shows the highest number and density of oxide nodules, see Figures S1–S3. With increasing exposure time, the surface coverage with oxide nodules generally increases. After 2000 h of exposure, AFA-A also shows a very high percentage of the fine ground surface covered by outward-growing Fe oxides. AFA-B shows the least amount of oxide nodules. Even after 5000 h, extended areas without nodules are present.

A closer look at the sample surfaces reveals that the outward-growing Fe oxide nodules are surrounded by a darker-appearing region (marked as (2) in Figure 4), which is depleted in Fe compared with the bulk material. Additionally, the dark regions show an oxygen content of 50–55 at%, which means that the AFA material is oxidized in these locations. The flat unaffected-looking surface areas (marked as (3) in Figure 4) also show elevated oxygen contents of around 25–30 at% when measured with 20 keV electrons, indicating the presence of a thin oxide layer. For more details on the various regions, the results of the cross-section analysis are presented below.

After 1000 h exposure to Pb at 500 °C, all three AFA materials show the formation of a thin Al-rich oxide scale on the fine ground surface, which protects the material from liquid Pb corrosion, see examples shown for AFA-A (Figure 5a) and AFA-B (Figure 6a). Neither selective dissolution of AFA alloying elements nor penetration of Pb is observed. In addition to the thin scale, much thicker oxide nodules are also observed in some locations, mainly where Nb-rich precipitates are found. The thick oxide nodules are composed of an outward-growing Fe oxide (XRD confirmed the presence of magnetite, see also Figure S4 in the Supplementary Material for the XRD pattern of AFA-A after 2000 h exposure), typically thicker and less extended, and an inward-growing mixed oxide with lower thickness but larger lateral extension. As for the thin Al-rich oxide scale, the oxide nodules are also protective against dissolution corrosion and Pb penetration. Although some Pb is found in the outward-growing oxide, no Pb is observed in the inward-growing part or in the bulk material below.

As the exposure proceeds, a higher fraction of the fine ground surfaces becomes covered with the thick multilayer scale, see results after 2000 h and 5000 h. After 2000 h (see Figure 5b and Figure 7a for typical images of AFA-A and AFA-C, respectively), the thick multilayer oxide scale has grown to a thickness of about 5–10 µm and is still protective against Pb penetration. Pb is neither found in the inward-growing oxidation region nor in the AFA material below. As shown in the EDX elemental line scans in Figure 5c and Figure 7b, the outward-growing Fe-based oxide (magnetite) also contains some Pb and Nb. The inward-growing mixed oxide region is composed of oxygen as well as of the AFA alloying elements Cr, Ni, Al, and Mn in a concentration similar to the AFA bulk materials, whereas Fe is strongly reduced compared with its bulk concentration. The oxygen content is lower than in the outward-growing oxide, which indicates that the mixed oxide region contains non-oxidized species, in particular Ni. Similar results for the composition of the inward-growing oxidation region are obtained for AFA-B after 2000 h and for AFA-A even after 5000 h in some regions.

After 5000 h, the thick oxide scale has spread over an even larger surface fraction. Nevertheless, there are still some regions covered by the thin Al-rich oxide scale that protects the AFA steels from dissolution corrosion and Pb penetration. Regarding the thick oxide scale, the inward-growing oxidation region has converted after 5000 h to a Cr-based oxide, which is enriched in Al and Mn, and still contains some Fe, but does not contain any Ni (see EDX line scans in Figure 6c,d and Figure 7d). This indicates that the non-oxidized species Ni have disappeared from that region, presumably via dissolution into the molten Pb. In most locations, the inward-growing oxide is penetrated by Pb, either homogeneously or locally (see SEM-BSE images, EDX line scans and EDX elemental mappings in Figure 5d, Figure 6b–f and Figure 7c–f). Pb is also found at the interface between oxide and bulk. Next to this interface, the AFA material is depleted in Ni and Cr, and locally penetrated by Pb. Ni/Cr depletion and Pb penetration into the bulk are most pronounced for AFA-C. The Ni depletion is attributed to selective dissolution into the molten Pb, whereas Cr depletion occurs due to the formation of the Cr-based thick oxide layer.

#### 3.2.2. Coarse Ground Surfaces

**Figure 8:**
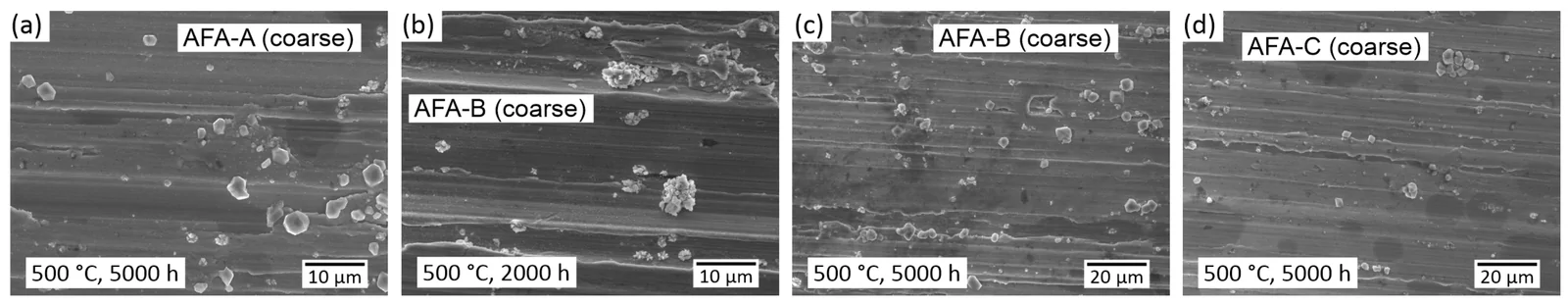
Coarse ground surfaces of AFA-A (**a**), AFA-B (**b**,**c**), and AFA-C (**d**) after exposure to liquid Pb at 500 °C as imaged by SEM-SE in surface view. Further images for other exposure times can be found in the Supplementary Material, Figure S5.

**Figure 9:**
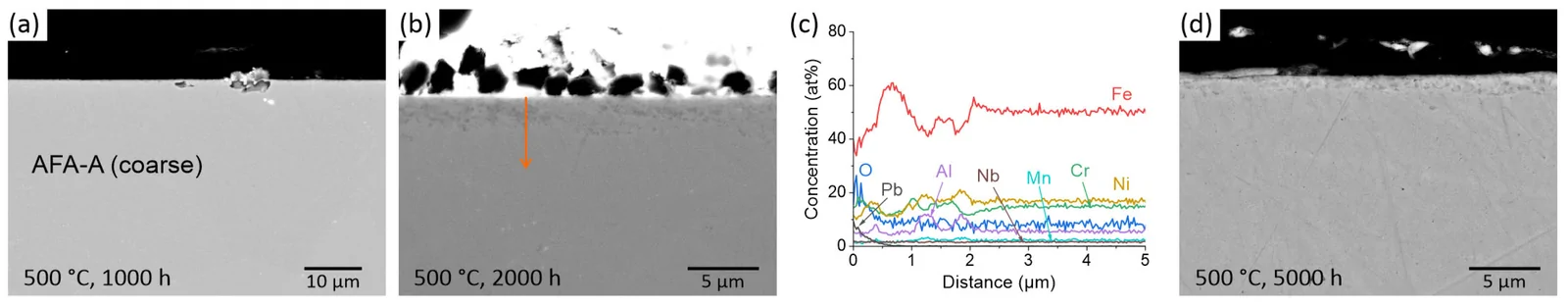
Cross-section analysis of coarse ground surface of AFA-A after exposure to liquid Pb at 500 °C. SEM-BSE images after 1000 h exposure (**a**), after 2000 h exposure (**b**) with corresponding result of EDX line scan (**c**), and after 5000 h exposure (**d**).

**Figure 10:**
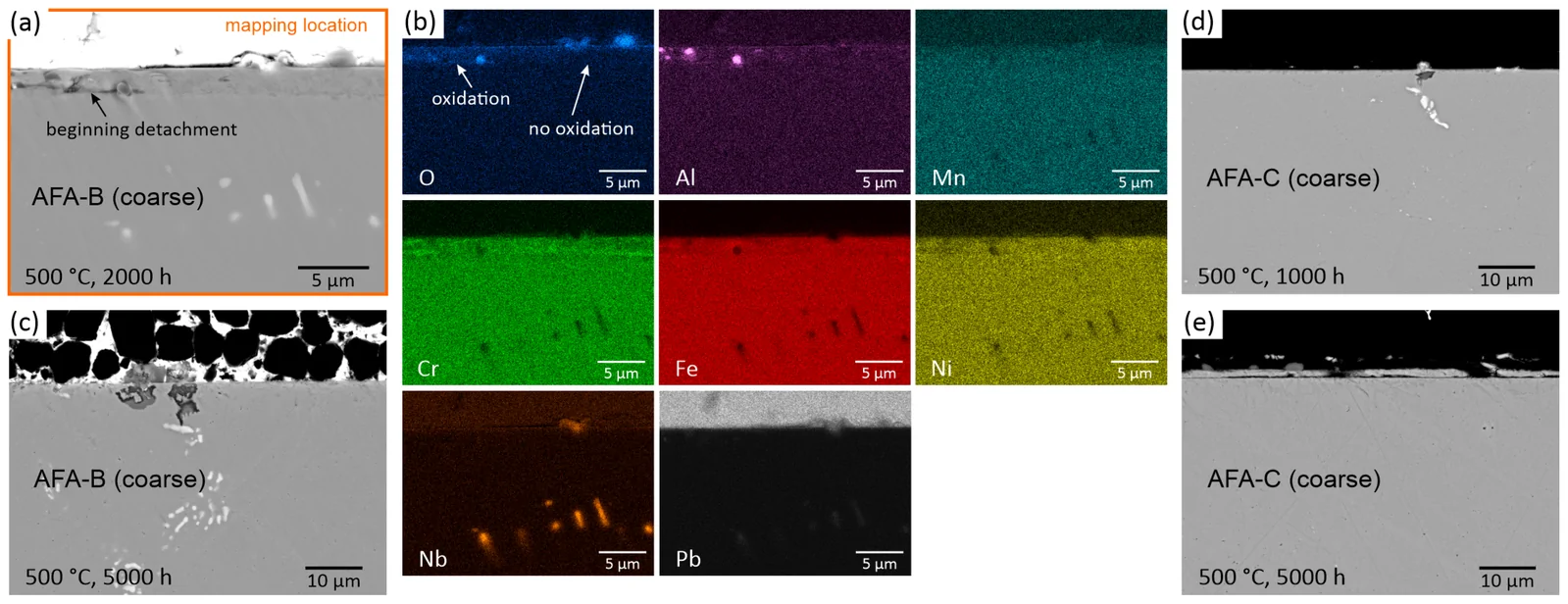
Cross-section analysis of coarse ground surfaces of AFA-B and AFA-C after exposure to liquid Pb at 500 °C. SEM-BSE images of AFA-B after 2000 h exposure (**a**) with result of EDX elemental mapping (**b**) and after 5000 h exposure (**c**), SEM-BSE images of AFA-C after 1000 h (**d**) and 5000 h exposure (**e**).

In contrast to the fine ground surfaces, the coarse ground surfaces of AFA-A, AFA-B, and AFA-C develop a thin and protective oxide scale during exposure to liquid Pb at 500 °C, see Figure 8, Figure 9 and Figure 10 and Figure S5. Although some oxide nodules are found on the surfaces (see surface images in Figure 8 and Figure S5 and examples shown in Figure 9a and Figure 10c,d in cross-section view), these nodules do not grow further and the oxide scale remains protective.

On all three AFA materials, the bulk directly below the thin oxide scale exhibits some microstructural changes. As shown in the EDX elemental line scan in Figure 9b,c, local enrichments of Ni and Al (NiAl formation, appears dark in BSE images) are observed next to Cr-enriched spots. These microstructural changes reach a depth of about 2 µm, independent of the exposure time.

With increasing exposure time, delamination of an around 2 µm thick layer of bulk material is observed, presumably caused by the described microstructural changes. An example is shown in Figure 10a,b, where the modified surface layer is still tightly attached to the bulk on the right side of the image but starts to detach on the left side. As indicated by the oxygen signal in Figure 10b, the newly built surface in the region with incipient detachment is already slightly oxidized, whereas no inner oxidation is found on the right. Further examples with remains of a detached surface layer are shown in Figure 9d for AFA-A and in Figure 10e for AFA-C. Despite delamination of the surface layer with the protective oxide scale on its surface, no liquid metal corrosion is observed during or after delamination on any of the AFA materials. This further confirms the excellent self-healing properties of the thin oxide scale on all three AFA materials when exposed to liquid Pb at 500 °C.

### 3.3. Corrosion at 600 °C

#### 3.3.1. Fine Ground Surfaces

The corrosion behavior of the fine ground surfaces of AFA-A, AFA-B, and AFA-C in molten Pb at 600 °C is summarized in Figure 11 and Figures S6–S8 in surface view and in Figure 12, Figure 13 and Figure 14 and Figure S9 in cross-section view.

**Figure 11:**
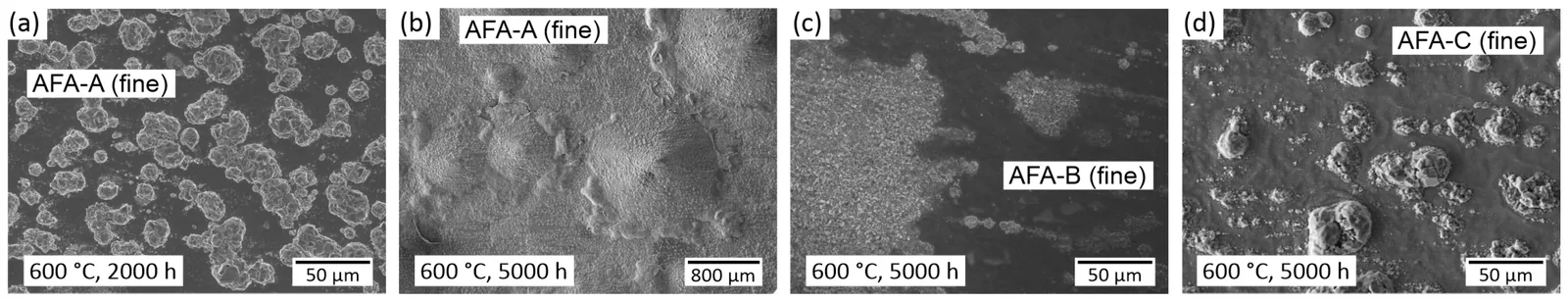
Fine ground surfaces of AFA-A (**a**,**b**), AFA-B (**c**), and AFA-C (**d**) after exposure to liquid Pb at 600 °C, imaged in surface view by SEM-SE. Further images for other exposure times can be found in the Supplementary Material, Figures S6–S8.

**Figure 12:**
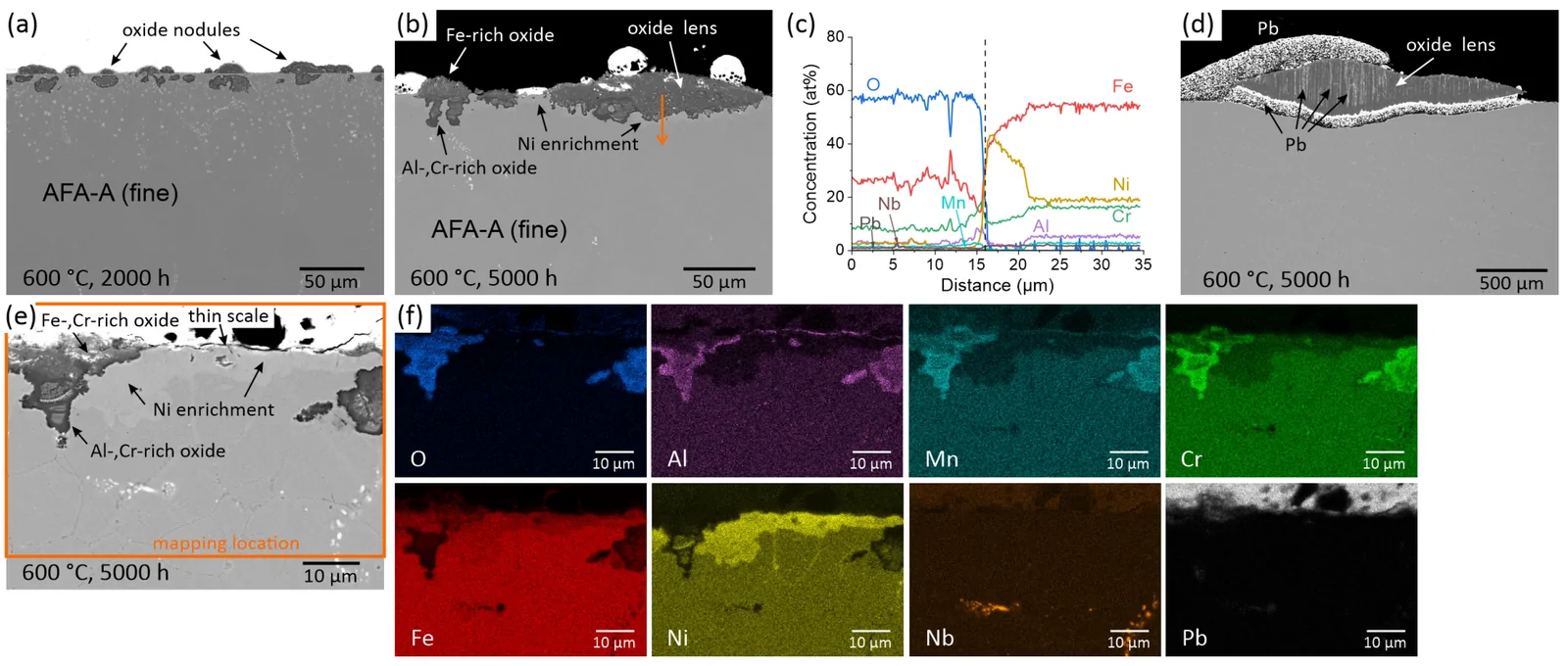
Cross-section analysis of fine ground surface of AFA-A after exposure to liquid Pb at 600 °C. SEM-BSE images after 2000 h (**a**) and 5000 h exposure (**b**,**d**,**e**). EDX elemental composition along line shown in (**b**) is presented in (**c**), EDX elemental mapping of (**e**) is shown in (**f**). The EDX elemental mapping result of (**b**) can be found in the Supplementary Material, Figure S9.

**Figure 13:**
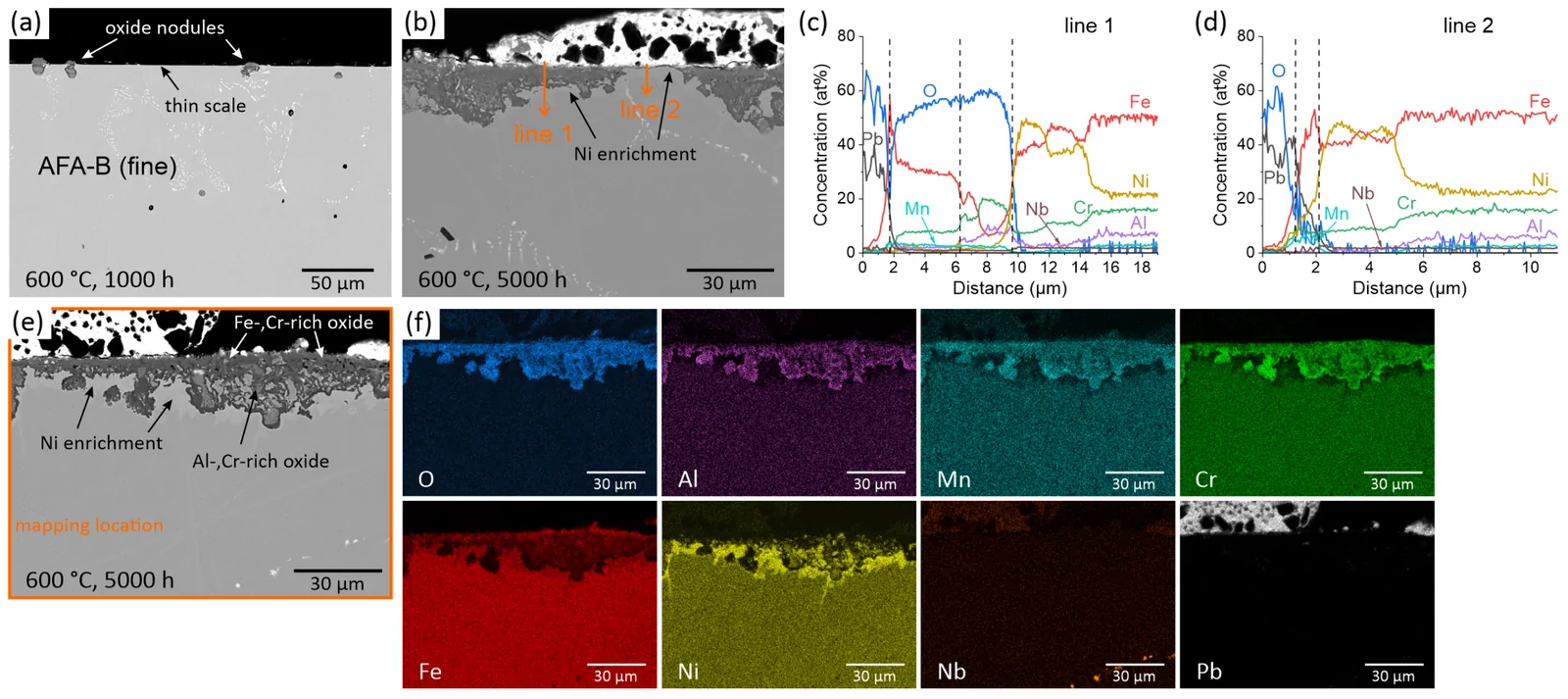
Cross-section analysis of fine ground surface of AFA-B after exposure to liquid Pb at 600 °C. SEM-BSE images after 1000 h (**a**) and after 5000 h exposure (**b**,**c**). EDX elemental compositions along lines shown in (**b**) are presented in (**c**,**d**), EDX elemental mapping of (**e**) is shown in (**f**).

**Figure 14:**
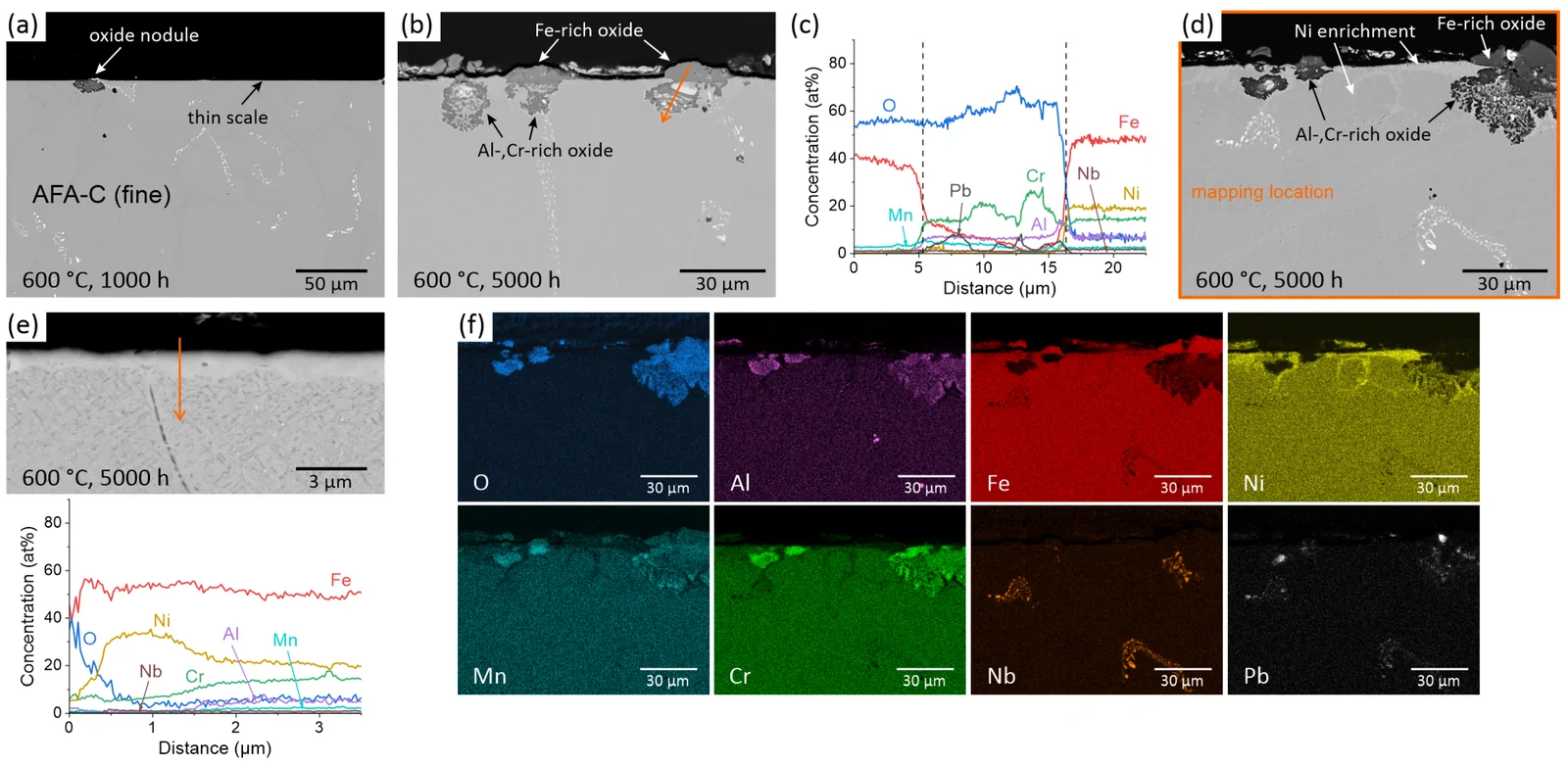
Cross-section analysis of fine ground surface of AFA-C after exposure to liquid Pb at 600 °C. SEM-BSE images after 1000 h (**a**) and 5000 h exposure (**b**,**d**,**e**). EDX elemental composition along line shown in (**b**) is presented in (**c**), EDX elemental mapping of (**d**) is shown in (**f**).

Similar to the exposure at 500 °C, at 600 °C a thin Al-rich oxide scale with very few thicker oxide nodules also covers the fine ground surfaces after 1000 h and protects the AFA samples from corrosion (see surface view in Figures S6–S8 and exemplary cross-sections in Figure 13a and Figure 14a). There is neither selective dissolution nor Pb penetration observed on any AFA material. The oxide nodules consist of an outward-growing Fe oxide (magnetite) and an inward-growing Cr-, Al-, and Mn-rich oxide (presumably spinel). With prolonged exposure, the number and size of thick oxide nodules increase (see Figure 11a and Figures S6–S8 for surface view images and Figure 12a for an exemplary cross-section after 2000 h) until, after 5000 h, extended regions of the fine ground surface are covered by a thick oxide layer and oxide nodules. Here, AFA-C shows the lowest surface fraction covered by thick oxides (oxide nodules only, see Figure 11d), whereas AFA-A and AFA-B show extended regions fully covered with thick oxide (Figure 11b,c) in addition to regions with separate oxide nodules (Figure 12d). The regions still covered by a thin oxide scale after 5000 h reveal the presence of Mn (in addition to Al) in the scale, see EDS elemental mapping in Figure 12f and line scan in Figure 13d. Furthermore, delamination at regions with the thin oxide scale is observed on AFA-B and AFA-C, see Figures S7 and S8.

In addition to the extent of thick oxide nodules/scale, their nature and composition also change with exposure time, which becomes apparent in the cross-section analysis. Here, three different oxide scale structures can be distinguished as follows. Firstly, the largest and most striking type of thick oxide scale structure is found on AFA-A only (Figure 12b–d). Here, in some locations, the multilayer oxide scale turns after 5000 h into a one-layer structure that grows both outward and inward, forming a symmetric “lens”. An example of a still rather small oxide lens is shown in Figure 12b (right nodule), whereas Figure 12d shows a much bigger oxide lens. Such oxide lenses appear to have a rotational symmetric structure, see the surface image of the respective region on the sample (Figure 11b). The oxide lenses contain Fe, Cr, Al, and Mn in a ratio that resembles the AFA bulk composition, see line scan in Figure 12b,c. Except for an enrichment of Cr and Al at the corrosion front, there is no inner structure or variation in composition found within the lenses. Ni is detected in the thick oxide lenses only in very small amounts (trapped Ni). Additionally, Pb is found to be incorporated into the particularly big oxide lenses (perpendicular bright stripes in Figure 12d). Below the oxide lens shown in Figure 12b, a clear Ni enrichment of the bulk material is observed, accompanied by a depletion in Cr, Al, and Mn (see line scan in Figure 12c and EDX elemental mapping in Figure S9). The Ni enrichment might be responsible for the Pb penetration below the large oxide lenses and their subsequent detachment as observed in Figure 12d.

The second type of thick oxide is observed on AFA-B (Figure 13b–f) and also in some locations of AFA-A (Figure 12e,f). The thick oxide scale is mainly growing inward and consists of two distinct regions, a surface-near region with a Fe-Cr-oxide and the main region composed of a Cr-, Al-rich oxide that is penetrated by Pb and Ni. The AFA bulk material in the respective regions is strongly enriched in Ni next to the described oxide scale (see mappings in Figure 12f and Figure 13f and line 1 in Figure 13b,c).

The third type of thick oxide is observed on AFA-C and in some locations of AFA-A, namely separate oxide nodules that still consist of an outward-growing Fe oxide (magnetite) and an inward-growing Cr- and Al-rich oxide, see left nodule in Figure 12b (corresponding mapping in Figure S9) for AFA-A and all nodules of AFA-C (Figure 14b–d). The inward-growing oxide shows the highest Cr and Al concentrations at the oxidation front, whereas Pb and (non-oxidized) Ni are incorporated in the central part of the inward-growing oxide nodules. Ni enrichment is observed below the thin oxide scale that still can be found between the oxide nodules and along the surface-near grain boundaries, see EDX elemental mapping in Figure 14f. Ni enrichment below the thin oxide scale is also demonstrated by the EDX elemental line scans shown in Figure 13b,d (line 2) and Figure 14e. In addition to the Ni enrichment, a depletion in Cr and a total absence of Al and Mn is observed in the 1–2 µm thick layer below the thin Al-, Mn-rich oxide scale. No/less Ni enrichment is found in the vicinity of the inward-growing oxides, probably due to the incorporation of Ni into these regions.

The Ni enrichment below the oxide scale (whether lens-type, multilayer, or thin) observed after exposure at 600 °C is in clear contrast to the exposure results at 500 °C, where Ni is not enriched but depleted below the oxide scale.

#### 3.3.2. Coarse Ground Surfaces

**Figure 15:**
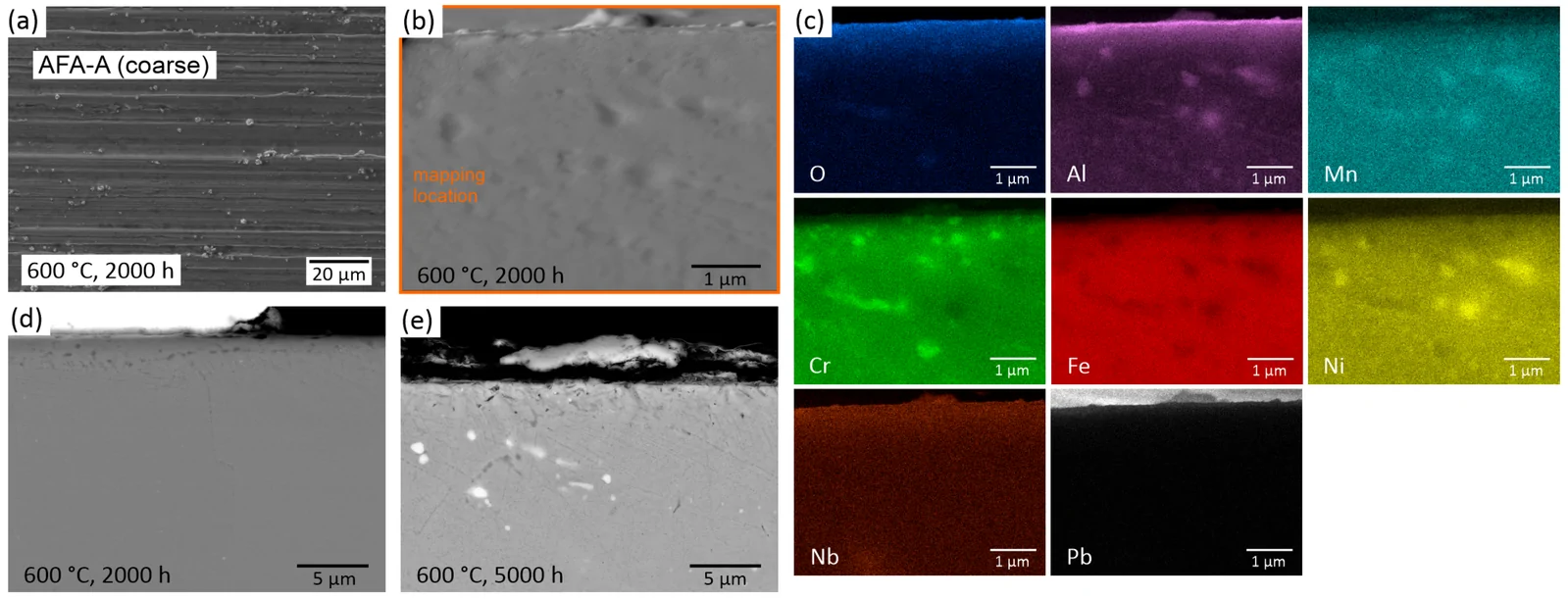
Coarse ground surface of AFA-A after exposure to liquid Pb at 600 °C. SEM-SE image of sample surface after 2000 h exposure (**a**), SEM-BSE cross-section images after 2000 h (**b**,**d**) and 5000 h exposure (**e**). EDX elemental mapping of (**b**) is shown in (**c**).

**Figure 16:**
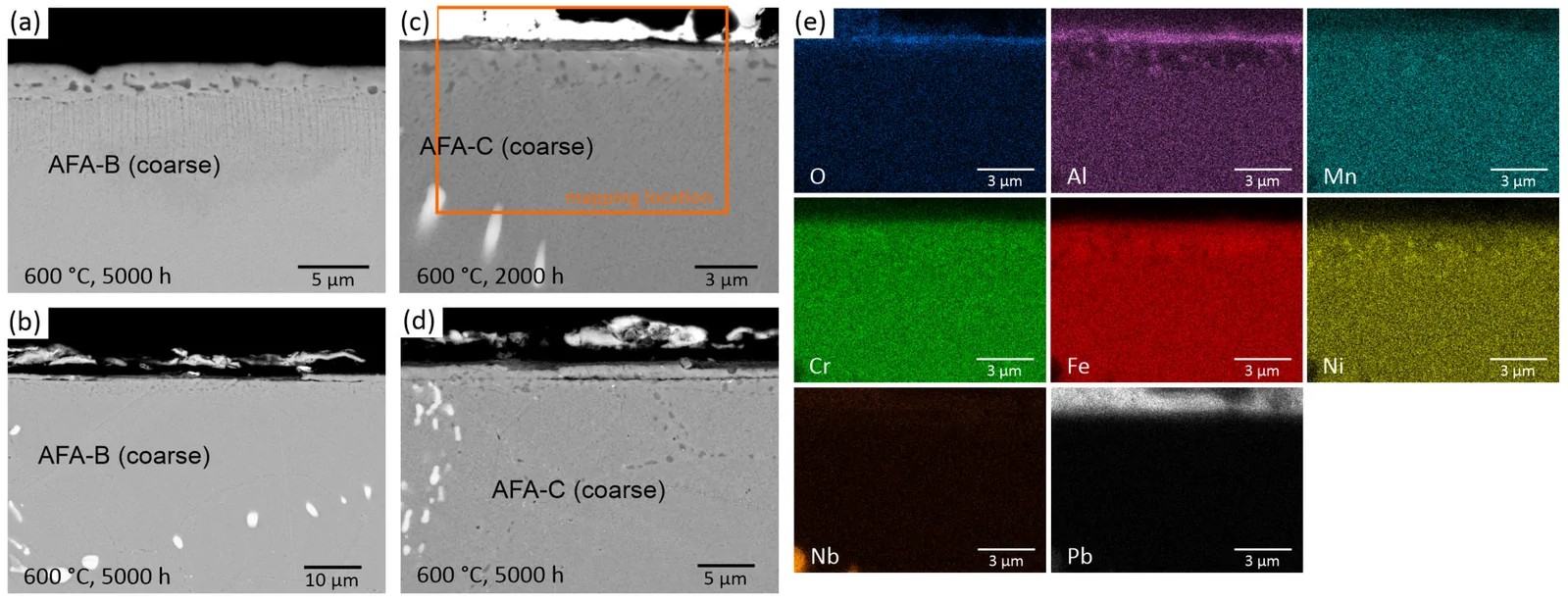
Cross-section analysis of coarse ground surfaces of AFA-B and AFA-C after exposure to liquid Pb at 600 °C. SEM-BSE images of AFA-B after 5000 h exposure (**a**,**b**). The mapping result of (**b**) can be found in the Supplementary Material, Figure S10. SEM-BSE images of AFA-C after 2000 h (**c**) and 5000 h exposure (**d**). The EDX elemental mapping of (**c**) is shown in (**e**).

The coarse ground surfaces of AFA-A, AFA-B, and AFA-C after exposure to liquid Pb at 600 °C are shown in Figure 15, Figure 16 and Figure S10. Similar to the coarse ground surfaces exposed at 500 °C, a thin and protective oxide scale forms at 600 °C. Below the oxide scale, NiAl formation and the development of Cr-rich spots is observed, see EDX elemental mapping in Figure 15c for an example of AFA-A. These microstructural changes are more pronounced than after exposure at 500 °C.

As for the exposure at 500 °C, at 600 °C delamination of the modified surface layer is also observed. In Figure 16b, remnants of delaminated material are found detached from the surface of AFA-B, whereas at the far left and the far right of the image, the surface layer is still attached to the surface. Similarly, Figure 16d shows incipient delamination for AFA-C and Figure 15e shows remnants of a detached surface layer for AFA-A. Despite the delamination, neither selective dissolution nor penetration of Pb is observed on the coarse ground surfaces after exposure at 600 °C.

Regarding the composition of the modified surface layer, distinct depletion in Al in the region between the thin Al-rich oxide scale and the spots with the NiAl secondary phase is observed on AFA-B (Figure S10) and on AFA-C, see EDX elemental mapping in Figure 16e. This depletion is mainly attributed to the Al consumption for oxide scale formation. Other elements such as Mn or Cr do not show any depletion in surface-near regions.

### 3.4. Corrosion at 700 °C

**Figure 17:**
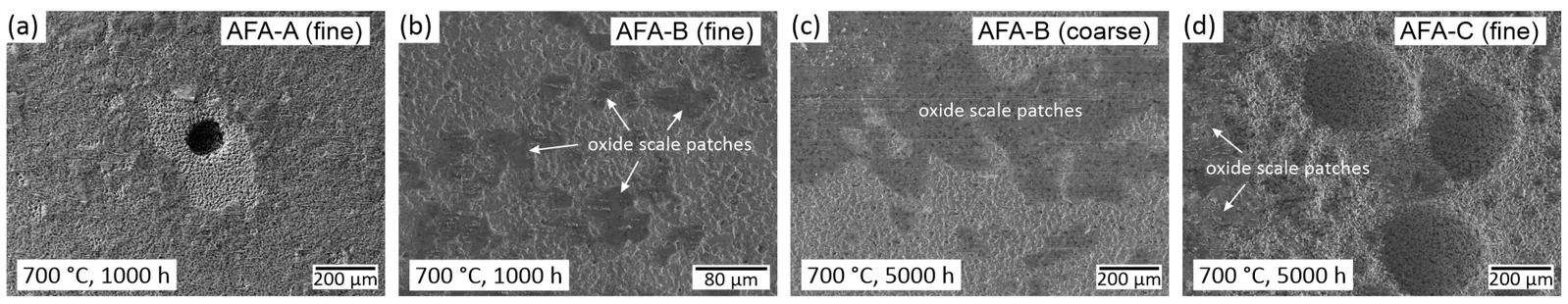
SEM-SE images of the surfaces of AFA-A (**a**), AFA-B (**b**,**c**), and AFA-C (**d**) after exposure to liquid Pb at 700 °C.

**Figure 18:**
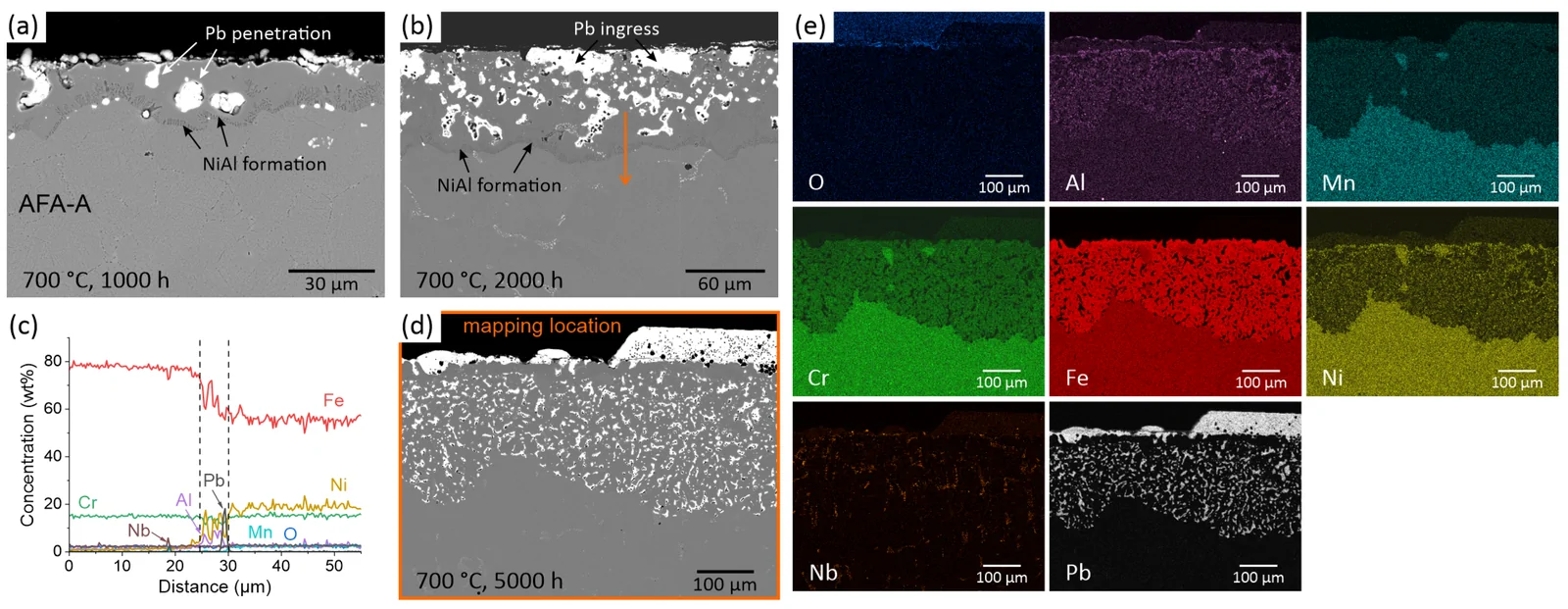
Cross-section analysis of AFA-A after exposure to liquid Pb at 700 °C. SEM-BSE images after 1000 h exposure (**a**), after 2000 h (**b**) with corresponding result of EDX line scan (**c**), and after 5000 h exposure (**d**) with corresponding result of EDX elemental mapping (**e**).

**Figure 19:**
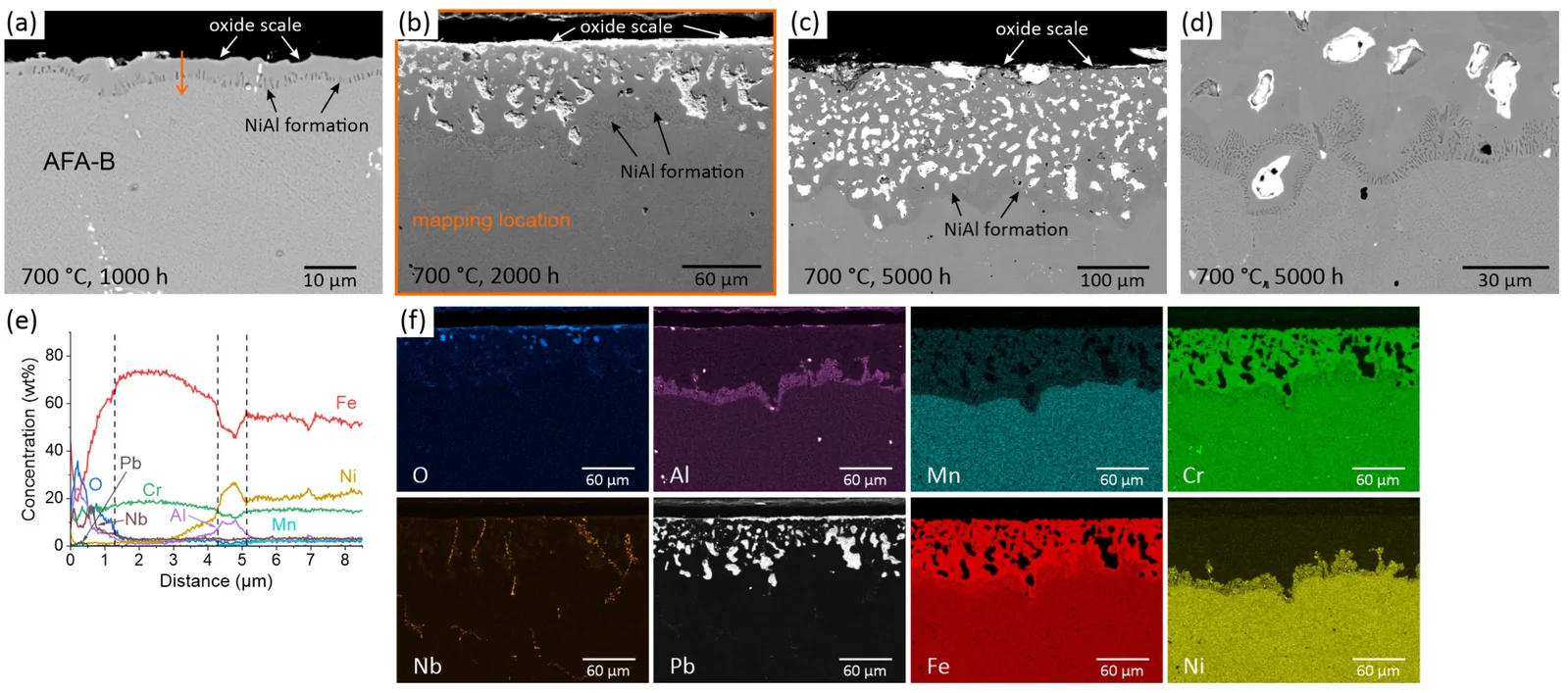
Cross-section analysis of AFA-B after exposure to liquid Pb at 700 °C. SEM-BSE image after 1000 h (**a**), SEM-SE image after 2000 h (**b**), and SEM-BSE images after 5000 h exposure (**c**,**d**). EDX elemental composition of line shown in (**a**) is presented in (**e**), EDX elemental mapping of (**b**) is shown in (**f**).

**Figure 20:**
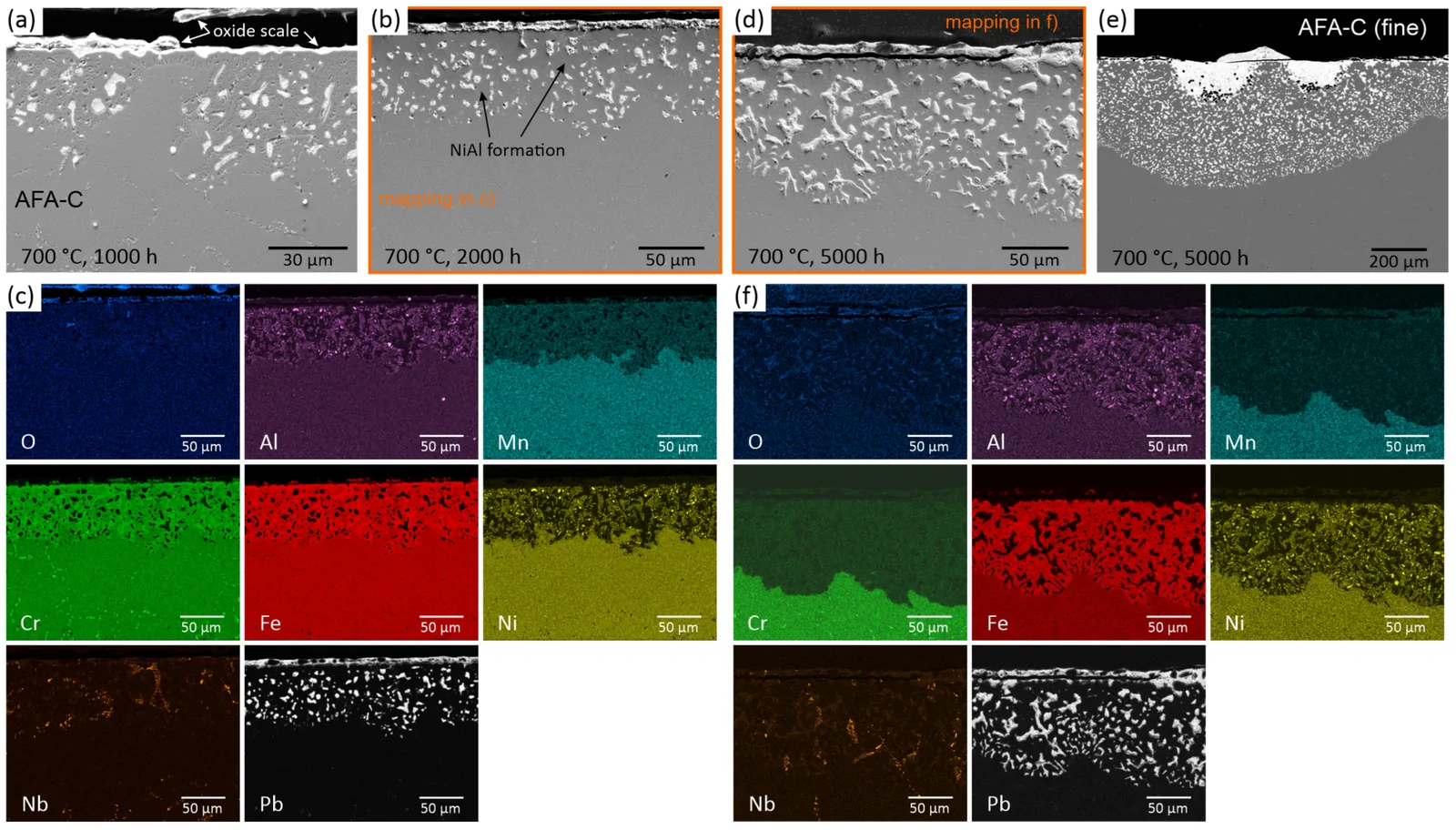
Cross-section analysis of AFA-C after exposure to liquid Pb at 700 °C. SEM-BSE image after 1000 h exposure (**a**), SEM-SE image after 2000 h (**b**) with corresponding EDX elemental mapping (**c**), SEM-SE image (**d**) and SEM-BSE image after 5000 h exposure (**e**) with EDX elemental mapping of (**d**) shown in (**f**).

All three AFA materials show liquid Pb corrosion after exposure at 700 °C, consisting of selective dissolution and Pb penetration, see Figure 17 for surface images and Figure 18, Figure 19, and Figure 20, respectively, for cross-section images of AFA-A, AFA-B, and AFA-C. The corrosion behavior presented below applies to both the fine and the coarse ground surfaces of the specimens. There is no systematic influence of the surface finish on the corrosion behavior observed at 700 °C.

The most severe corrosion features found after exposure at 700 °C are rather large circular holes in the specimens of AFA-A and AFA-C as exemplarily shown in Figure 17a,d and Figure 20e. These holes are found preferentially (but not only) on the fine ground surfaces. In these locations, the AFA material was totally dissolved into the adjacent liquid Pb. The holes are surrounded by a large volume showing selective dissolution of Ni, Cr, and Mn, and penetration of Pb. It seems that the holes have formed in locations where a corrosion-mitigating oxide scale failed very early during exposure or has not formed at all. The failure or absence of an oxide scale allowed dissolution of all alloying elements without any barrier, which resulted in the observed holes. From this behavior, it can be concluded that in locations without holes and/or with lower corrosion depths, the dissolution process must have been slowed down—at least in the early state of exposure—by some kind of barrier, i.e., by an oxide scale on the sample surface. As presented by the surface images in Figure 17, an oxide scale is indeed found on all three AFA materials. After 1000 h, the fine ground surface of AFA-A is almost entirely covered by an oxide scale (entire surface shown in Figure 17a except for the brighter area surrounding the hole), whereas oxide scale patches are found on the fine ground surface of AFA-B (Figure 17b). Even after 5000 h of exposure, darker-appearing patches of an oxide scale are found on the sample surfaces, see Figure 17c,d for examples of AFA-B (coarse ground) and AFA-C (fine ground). EDX point measurements on the dark patches show oxygen contents between 35 and 60 at% depending on the accelerating voltage (20 to 10 keV), which confirms the formation of a thin oxide layer. Furthermore, the Al content is clearly enhanced compared with its bulk value, confirming the formation of an Al-rich oxide.

From the cross-section analysis of the specimens after exposure to liquid Pb at 700 °C, it is found that after 1000 h of exposure, a corrosion layer of 5–7 µm thickness similar to the one shown in Figure 19a for AFA-B is formed on all three AFA materials. As seen in the EDX elemental line scan result in Figure 19e, the corrosion layer is depleted in Ni and Mn, while an Al-rich oxide scale is found at the sample surface. The Ni and Mn depletion below the oxide scale indicates non-protective properties of the scale, allowing Ni and Mn to dissolve into the molten Pb. At the corrosion front, i.e., at the interface between the corrosion layer and the non-affected AFA bulk material, the formation of a secondary phase (NiAl) is observed in the corroded region.

In addition to the comparably thin corrosion layer of 5–7 µm thickness just described, all three AFA materials also show regions with deeper corrosion already after 1000 h, see Figure 18a and Figure 20a for examples. Here, the corrosion depths reach 25 µm on AFA-A, 90 µm on AFA-B, and 65 µm on AFA-C. As for the thin corrosion layer, the regions with deeper attack also exhibit a thin Al-rich oxide scale at the surface (though not continuous), a Ni- and Mn-depleted corroded zone, and NiAl formation at the corrosion front. Additionally, Pb penetration into the corrosion region is observed.

After 2000 h of exposure to liquid Pb at 700 °C, the corrosion layers of AFA-A, AFA-B, and AFA-C reach larger depths compared with the 1000 h exposure, see Figure 18b, Figure 19b and Figure 20b. As clearly observed in the EDX elemental mapping of AFA-B (Figure 19f), the surface is still covered by a thin Al-rich oxide scale, which also contains Pb. The corrosion-affected zone below the oxide scale is depleted in Ni and Mn, enriched in Fe, and penetrated by Pb, see also line scan in Figure 18b. AFA-A additionally shows locations with stronger Pb ingress, most probably due to local failures of the oxide scale (e.g., spallation). The Cr content of the corrosion region is either slightly enriched compared with the bulk (AFA-B, see mapping in Figure 19f) or similar to the bulk (AFA-A, see line scan in Figure 18b). For both AFA-A and AFA-B, the NiAl secondary phase formation remains localized in a stripe along the corrosion front. In contrast, AFA-C shows NiAl formation in a much broader band, extending in some regions over in the entire corrosion zone (Figure 20b,c). Nevertheless, the Cr content of the matrix between the NiAl secondary phase is still slightly enriched compared with its bulk concentration.

After 5000 h, there are still patches of the surface covered with an intact thin oxide scale. In addition, local failures of the oxide scale with stronger Pb ingress are observed for all materials, and the maximum corrosion depth increases further to 280 µm for AFA-A, 225 µm for AFA-B, and 200 µm for AFA-C, cf. Figure 18d, Figure 19c and Figure 20d,e. For AFA-B, NiAl formation is still restricted to a thin stripe along the corrosion front, see Figure 19c,d. The corrosion region is depleted in Ni and Mn, while the Cr content of the corrosion region is similar to the bulk material (see corresponding EDX line scan results). AFA-A and AFA-C show corrosion layers with the NiAl secondary phase formation extending over (almost) the entire corroded zone, see Figure 18d,e and Figure 20d,f. This behavior resembles the observations for AFA-C after 2000 h (Figure 20b,c). However, in contrast to the results of AFA-C after 2000 h, the corroded region after 5000 h is depleted in Cr for AFA-A (9 wt% remain in the Fe-rich matrix) and strongly depleted in Cr for AFA-C (2 wt% in Fe-rich matrix), see also mappings in Figure 18e and Figure 20f. This indicates that the oxide layer became permeable not only for Ni and Mn but also for Cr or entirely disappeared. From the differences in corrosion behavior observed after 5000 h exposure at 700 °C, it can be concluded that the quality and/or durability of the oxide scale is best for AFA-B (the least amount of Cr is lost), medium for AFA-A, and worst for AFA-C. For all three materials, the corroded regions are penetrated by Pb.

## 4. Discussion

**Figure 21:**
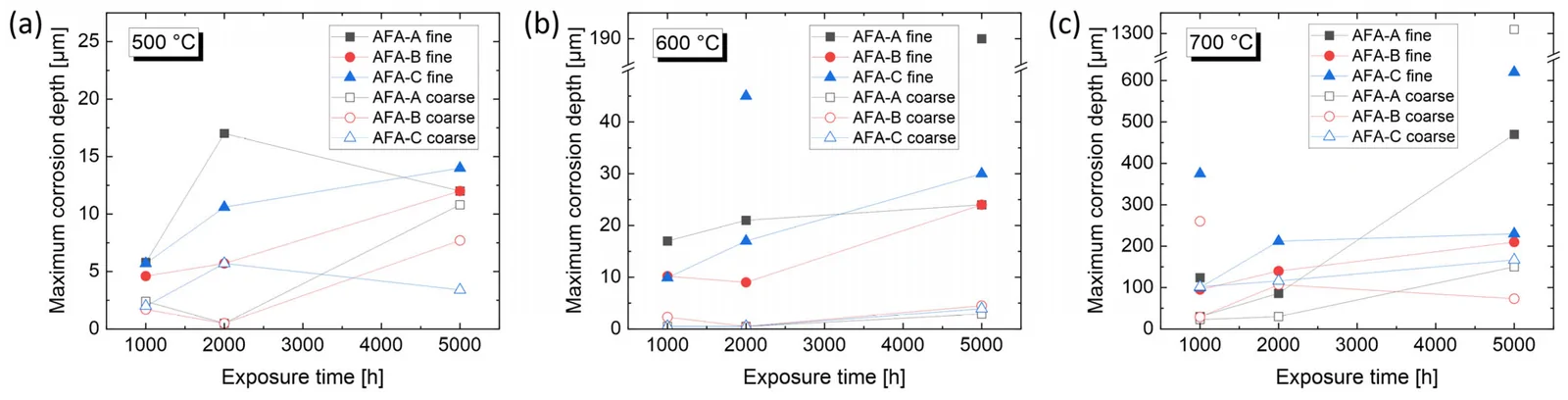
Maximum corrosion depth versus exposure time for both the fine ground and the coarse ground surfaces of AFA-A, AFA-B, and AFA-C. The data for 500 °C (**a**) and 600 °C (**b**) show the maximum penetration depth of the inward-growing thick oxide into the material, including Pb penetration and dissolution below the scale where applicable. The data for 700 °C (**c**) show the maximum depth of the dissolution attack. Large anomalies are shown as separate data points not connected with the other data.

All three Fe24Ni15Cr3Al-based AFA materials investigated in the present study show good corrosion resistance up to 600 °C, whereas dissolution attack is found at 700 °C. This agrees with [28], which reports excellent corrosion resistance at 600 °C but beginning dissolution at 650 °C for an AFA steel with similar composition. At 500 and 600 °C, a clear influence of the surface finish is observed in the present study. Coarse ground surfaces exhibit superior corrosion resistance thanks to the formation of a thin Al-rich oxide scale, whereas fine ground surfaces show enhanced oxidation and the formation of a thick multilayer oxide scale susceptible to Pb penetration. To summarize the observed corrosion behavior, Figure 21 shows the maximum corrosion depth versus exposure time for the different materials, surface finishes, and exposure temperatures. Despite the large scatter in the data, the general trends described so far are reproduced. These trends regarding the influence of the surface finish, the exposure temperature, and the differences in material composition on the corrosion behavior are discussed below in more detail.

### 4.1. Surface Finish

All AFA specimens were prepared with one fine ground surface and one coarse ground surface. Although the grinding was performed manually without any precise control of the grinding pressure and duration, the observed corrosion behavior shows a statistically significant difference between the coarse and the fine ground surfaces. Both at 500 °C and at 600 °C, all fine ground surfaces show the formation of thick oxide scales, whereas all coarse ground surfaces are protected by thin oxide scales, interspersed merely with some individual oxide nodules that do not deteriorate the corrosion resistance. This is also reflected by the maximum corrosion depth shown in Figure 21. Although the data of the coarse ground surfaces exposed at 500 °C include the oxide nodules, their penetration into the material remains below the corrosion depth of the fine ground surfaces (Figure 21a). The influence of the surface finish becomes even more striking after exposure at 600 °C, see Figure 21b. A closer look at the AFA material below the thin oxide scales reveals the formation of NiAl on the coarse ground sides. Examples of NiAl formation, in some cases accompanied by the appearance of Cr-rich spots, in the subsurface layer below a thin Al-rich oxide scale are also found in the literature for AFA materials exposed to liquid Pb/LBE in the temperature range 550–600 °C [18,19,23,25,28]. Although the fine ground surfaces also show regions with thin oxide scales, NiAl formation below the thin scale is less pronounced or not observed on the fine ground sides, see examples in Figure 12e and Figure 14e. Since the rearrangement of alloying elements and microstructural changes accompanied with the NiAl formation on the coarse ground surfaces (along with their absence on the fine ground surfaces) extend to a depth of about 2 µm, they cannot be induced by the difference in surface topography only. A reasonable explanation is an enhanced mobility of alloying elements in the respective subsurface layer of the coarse ground sides compared with the subsurface layer of the fine ground sides, presumably due to a higher density of dislocations generated during grinding with the coarse-grained SiC paper. It is known that dislocations generated by surface treatments such as grinding or turning can lead to recrystallization and grain refinement in the subsurface layer, accompanied by enhanced diffusion [17,27]. The higher mobility and enhanced diffusion also explain the better ability to form a thin Al-rich oxide scale and to heal that scale once a thicker oxide nodule forms due to a defect or precipitate in the material.

At 500 °C and 600 °C, protective oxide scales with self-healing properties form on the coarse ground surfaces only, whereas the thick oxide scales formed on the fine ground surfaces are susceptible to Pb penetration for prolonged exposure. Therefore, it is recommended to “activate” the surface/subsurface of materials and components by coarse-grained grinding or some other cold working surface finish prior to exposure to liquid Pb. Some researchers even recommend activating the entire bulk material by cold working, which was demonstrated to substantially enhance the diffusion of solute alloying elements (Al, Cr) and thus support the growth of an Al-rich oxide scale with sustained protective properties [27].

The influence of the surface finish on the corrosion behavior is much less pronounced, if present at all, at 700 °C, where selective dissolution and Pb penetration are observed in addition to the formation of a thin oxide scale (Figure 21c). This is explained by the generally enhanced kinetics of all involved competing processes at the elevated temperature, including the diffusion processes in the AFA materials. The small differences in mobility induced by the different grinding processes lose their relevance at the higher temperature of 700 °C.

### 4.2. Exposure Temperature

The exposure tests were performed at three different temperatures. At 500 and 600 °C, the AFA materials show good corrosion resistance thanks to the formation of an oxide scale, whereas severe corrosion including dissolution and Pb penetration is observed at 700 °C. Notably, the corrosion depths observed at 700 °C are much larger than the material recession caused by the growth of thick oxides at 500 and 600 °C, see Figure 21 and the different scales on the y-axis. It is well known that above some critical temperature the dissolution process predominates the oxide formation. Besides the enhanced diffusion and oxidation kinetics at increased temperature, one of the main factors is the higher solubility of alloying elements in liquid Pb. Another parameter is the oxygen activity. Since in the present experimental campaign, the concentration of dissolved oxygen (1 × 10^−7^ wt.%) was the same at all tested temperatures, the oxygen activity was lowest at the highest temperature. Oxidation potentials, on the other hand, increase with temperature. This makes the formation of oxides thermodynamically less favorable at higher temperatures. It might be possible to obtain much better corrosion resistance at 700 °C when using an increased oxygen content to achieve at least the same oxygen activity as at 600 °C.

Regarding the oxide scales formed at the lower temperatures, there are some important differences observed between the results at 500 °C and the ones at 600 °C. On the fine ground surfaces, Ni is depleted below the oxide scale at 500 °C, whereas a Ni enrichment below the scale is obtained at 600 °C. Furthermore, the inward-growing oxide scale is infiltrated by Pb and Pb is found below the scale at 500 °C, whereas Pb penetration into or below the scale is hardly observed at 600 °C. Both observations clearly indicate that the scale grown at 600 °C is more protective than the scale formed at 500 °C, i.e., it blocks diffusion of both Ni and Pb more efficiently. It is known that Al oxidizes to θ-Al_2_O_3_ at lower temperature, whereas growth of the denser and more stable α-Al_2_O_3_ occurs at higher temperature. Similarly, mixed oxides tend to form a denser structure at higher temperature, for instance, due to a higher Cr content in a Fe-Cr spinel. In the present case, it is found that the Fe oxide grown outward at 600 °C contains more Mn than the one formed at 500 °C, which improves its density and stability.

### 4.3. Material Composition

Finally, the material-specific differences of the AFA materials investigated in the corrosion behavior are discussed. Three different AFA materials have been investigated, which vary slightly in their Al, Cr, and Ni contents and which contain small amounts of either V or W, see Table 1. Because the material composition variations are minor, it is not a surprise that the differences in corrosion behavior are also minor.

At 500 °C, AFA-B shows the least fraction of the fine ground surface covered by thick oxide nodules after 5000 h exposure, i.e., the highest surface fraction is covered by a thin scale, which indicates a slightly better corrosion resistance of AFA-B compared with the other two AFA materials. At regions with thick oxide scale, AFA-B also shows slightly lower oxidation depths (Figure 21a), while Ni/Cr depletion below the scale and Pb penetration into the bulk are most pronounced for AFA-C, indicating slightly inferior corrosion resistance of AFA-C. Thus, AFA-B performs best at 500 °C, closely followed by AFA-A and AFA-C, see summarized assessment of corrosion resistance in Table 2. The superior behavior of AFA-B over AFA-A might be related with its slightly higher Al content, while the slightly lower Cr content of AFA-C may explain the inferior behavior of AFA-C. The corrosion behavior of the coarse ground surfaces does not show any differences between AFA-A, AFA-B, and AFA-C at 500 °C.

The fine ground surfaces show the following differences after exposure at 600 °C. AFA-A is the only material that forms large-scale oxide lenses after 5000 h (see also anomalous data points in Figure 21b). The least surface fraction covered with thick oxide is found for AFA-C. The type of thick scale varies. AFA-C forms separate oxide nodules, AFA-B exhibits laterally extended inward-growing oxides, and AFA-A shows both features plus the large-scale lenses. Thus, AFA-C performs best at 600 °C, possibly thanks to its higher Al content, closely followed by AFA-B. AFA-A with the lowest Al content shows clearly the worst corrosion behavior at 600 °C (cf. Table 2).

Coarse ground surfaces exposed at 600 °C exhibit surface-near restructuring with NiAl formation on all three materials. Within the restructured surface layer, Cr-rich spots are observed for AFA-A only, whereas Al depletion in the matrix is observed for AFA-B and AFA-C but not for AFA-A. It is not clear whether these differences are advantageous or disadvantageous for corrosion resistance.

At 700 °C, AFA-A and AFA-C show big holes formed by dissolution. The absence of such holes for AFA-B makes it the material with the best corrosion resistance at 700 °C. Furthermore, the least amount of Cr is lost from the corrosion layer of AFA-B, whereas the highest amount of Cr is dissolved for AFA-C. This further confirms the best performance of AFA-B and indicates a slightly better corrosion resistance of AFA-A compared with AFA-C at 700 °C as summarized in Table 2. In addition, AFA-C was found to form rather large sigma phase precipitation along the grain boundaries after exposure at 700 °C, which might have a negative influence on its mechanical properties.

To conclude, AFA-B exhibits the best corrosion resistance in the temperature range of 500–700 °C among the AFA materials tested in the presented study. It neither forms large oxide lenses at 600 °C nor large dissolution holes at 700 °C.

## 5. Conclusions

Three different Fe24Ni15Cr3Al-based AFA steels (AFA-A, AFA-B, AFA-C), developed and manufactured within the INNUMAT project, have been exposed to oxygen-controlled liquid Pb at 500 °C, 600 °C, and 700 °C for up to 5000 h to investigate their oxidation/corrosion behavior. For the same dissolved oxygen concentration of 1 × 10^−7^ wt.% at each temperature, the following conclusions can be drawn from the results of the exposure tests.

- The AFA steels retain their austenitic phase without significant grain growth even after 5000 h at 700 °C. However, large NbC precipitates are obtained and intergranular NiAl formation intensifies for higher exposure temperature.
- All three AFA steels show good corrosion resistance with oxide scale formation for temperatures up to 600 °C but severe dissolution corrosion and Pb penetration at 700 °C. The corrosion resistance at 700 °C might be improved by using a higher oxygen content to achieve at least the same oxygen activity as at 600 °C.
- At 500 °C and 600 °C, thin Al-rich oxide scales grow on the coarse ground surfaces of all investigated AFA steels, while enhanced oxidation and the formation of thick multilayer oxide scales are observed on the fine ground surfaces of the AFA materials. The superior oxidation/corrosion resistance of coarse ground surfaces is attributed to an enhanced elemental mobility due to a higher concentration of dislocations.
- The oxide scales formed at 600 °C are more protective against Ni dissolution and Pb penetration than the scales formed at 500 °C.
- Among the tested AFA steels, AFA-B shows the best corrosion resistance. The slightly inferior corrosion behavior of AFA-A is attributed to its lower Al content, while the presence of W in AFA-C promotes the precipitation of an unwanted sigma phase.

Regarding improvements in the AFA compositions, it is recommended to reduce the Nb and C contents to minimize the formation of large carbides. The influence of intergranular NiAl and carbide formation on the mechanical properties needs to be evaluated in future work.

## Figures and Tables

**Table 1:** Measured composition of AFA materials as provided by the manufacturer [wt%].

	C	Cr	Ni	Al	Mn	Nb	V	W	Y	Fe
AFA-A	0.076	14.91	22.67	2.6	3.03	0.75	0.19	-	0.01	Bal.
AFA-B	0.077	14.86	24.96	3.1	3.04	0.74	0.19	-	0.01	Bal.
AFA-C	0.082	14.1	25.25	3.8	3.03	0.72	-	1.04	0.01	Bal.

**Table 2:** Assessment of material-specific differences in corrosion resistance.

Exposure Temperature	Corrosion Resistance
500 °C	B>A>C
600 °C	C>B≫A
700 °C	B≫A>C

## Data Availability

The original contributions presented in this study are included in the article/Supplementary Material. Further inquiries can be directed to the corresponding author.

## Supplementary Materials

The following supporting information can be downloaded at https://www.mdpi.com/article/10.3390/ma19183826/s1. Figure S1: Fine ground surface of AFA-A after exposure to liquid Pb at 500 °C, imaged in surface view by SEM-SE; Figure S2: Fine ground surface of AFA-B after exposure to liquid Pb at 500 °C, imaged in surface view by SEM-SE; Figure S3: Fine ground surface of AFA-C after exposure to liquid Pb at 500 °C, imaged in surface view by SEM-SE; Figure S4: XRD pattern of fine ground surface of AFA-A after 2000 h exposure to liquid Pb at 500 °C; Figure S5: Coarse ground surfaces of AFA-A (a–c), AFA-B (d–f), and AFA-C (g–i) after exposure to liquid Pb at 500 °C, imaged in surface view by SEM-SE; Figure S6: Fine ground surface of AFA-A after exposure to liquid Pb at 600 °C, imaged in surface view by SEM-SEM; Figure S7: Fine ground surface of AFA-B after exposure to liquid Pb at 600 °C, imaged in surface view by SEM-SE; Figure S8: Fine ground surface of AFA-C after exposure to liquid Pb at 600 °C, imaged in surface view by SEM-SE; Figure S9: SEM-BSE cross-section image of fine ground surface of AFA-A after 5000 h exposure to liquid Pb at 600 °C (a) with corresponding EDX elemental mapping (b); Figure S10: Cross-section analysis of coarse ground surface of AFA-B after exposure to liquid Pb at 600 °C. SEM-BSE images after 2000 h (a) and 5000 h exposure (b,c) with EDX elemental mapping of location shown in (c) presented in (d).
