# Supplementary material for: Fe24Ni15Cr3Al-Based AFA Steels in Oxygen-Controlled Liquid Pb at 500 °C, 600 °C, and 700 °C
Source: Materials (Basel). 2026 Sep 8;19(18):3826. doi: 10.3390/ma19183826 (PMC13608718; doi:10.3390/ma19183826)
Supplement: Supplementary file 1 [file materials-19-03826-s001.zip › materials-4527026-supplementary.pdf]

Fe24Ni15Cr3Al-based AFA steels in oxygen-controlled liquid Pb at 500 °C, 600 °C, and 700 °C

Renate Fetzer*, Annette Heinzel, Alfons Weisenburger, Georg Müller

Institute for Pulsed Power and Microwave Technology (IHM), Karlsruhe Institute of Technology (KIT), Hermann-von-Helmholtz-Platz 1, 76344 Eggenstein-Leopoldshafen, Germany

*Corresponding author. E-Mail address: renate.fetzer@kit.edu

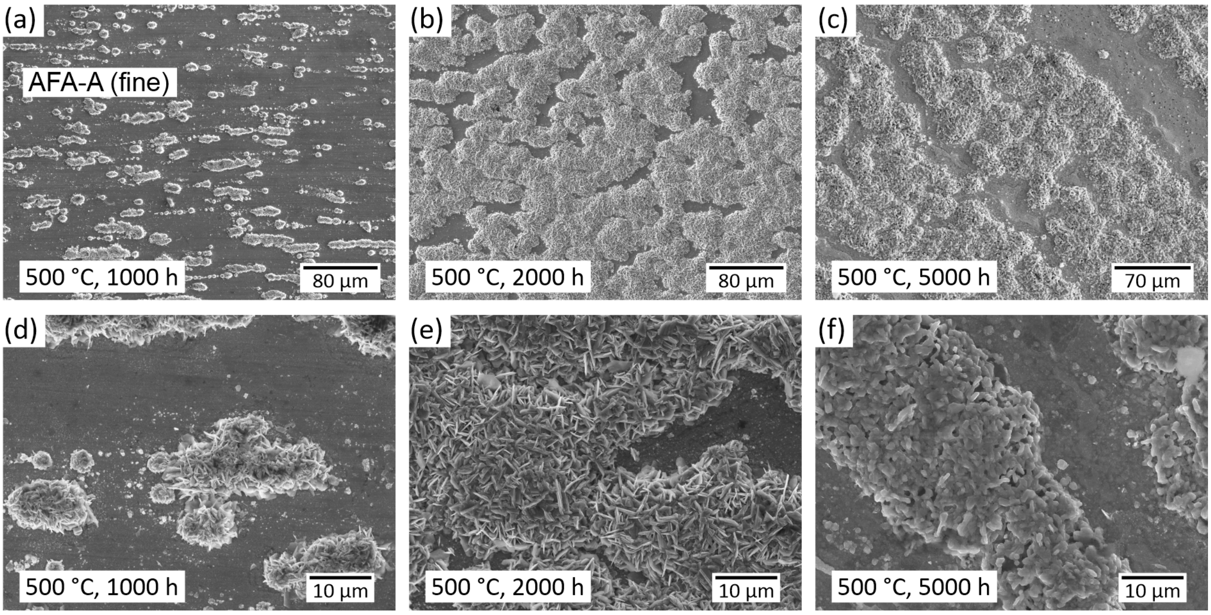

Figure S1: Fine ground surface of AFA-A after exposure to liquid Pb at 500 °C, imaged in surface view by SEM-SE.

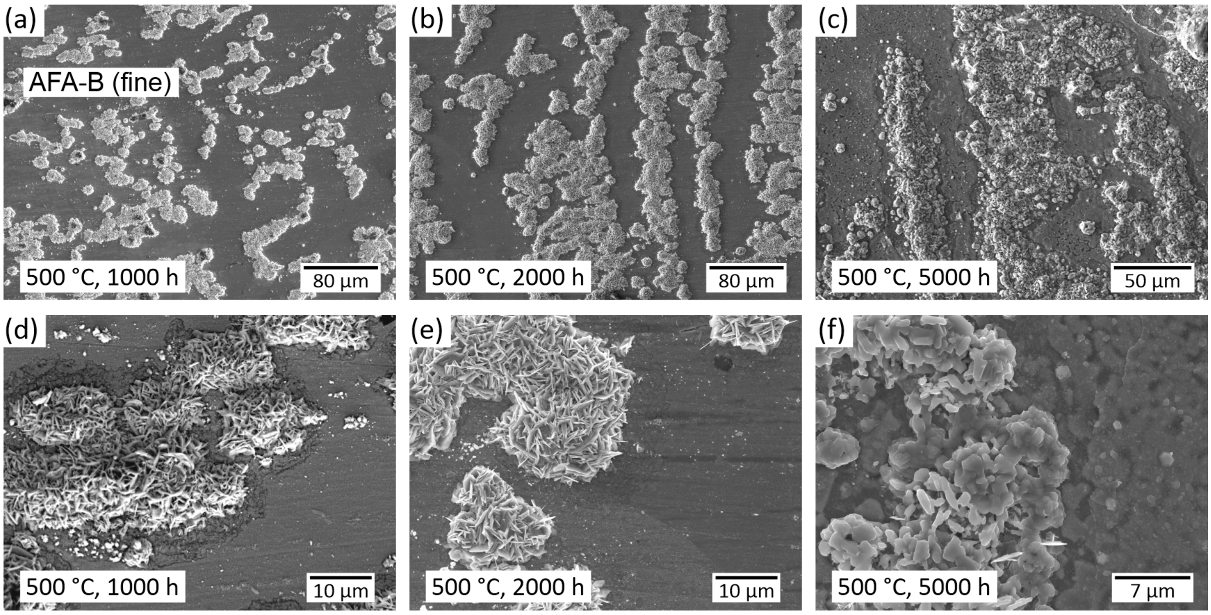

Figure S2: Fine ground surface of AFA-B after exposure to liquid Pb at 500 °C, imaged in surface view by SEM-SE.

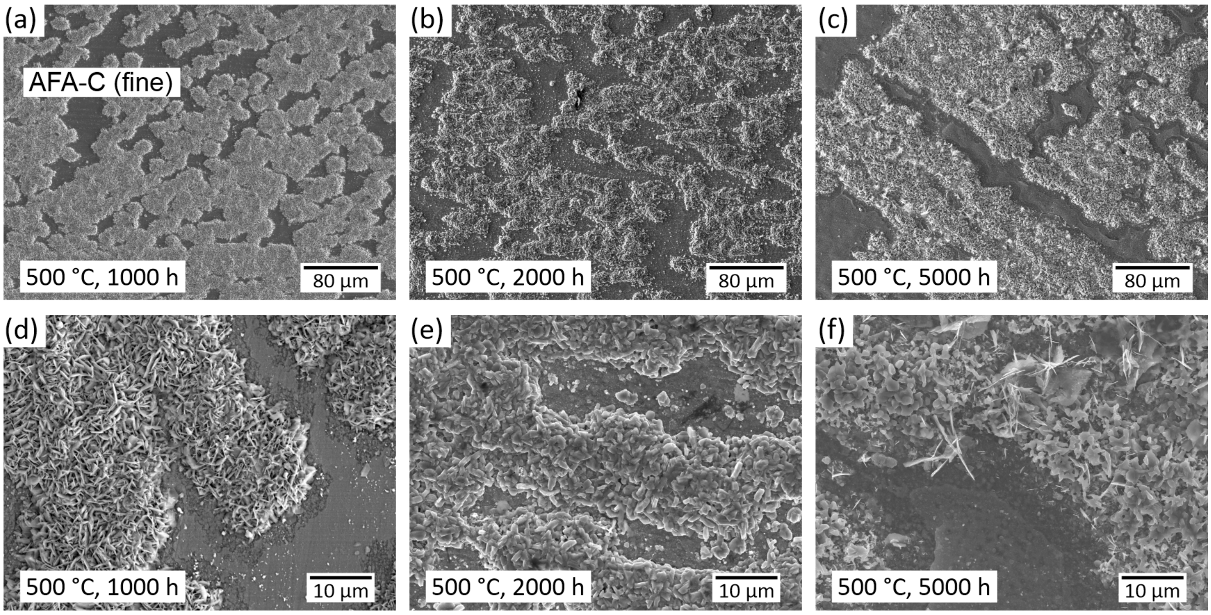

Figure S3: Fine ground surface of AFA-C after exposure to liquid Pb at 500 °C, imaged in surface view by SEM-SE.

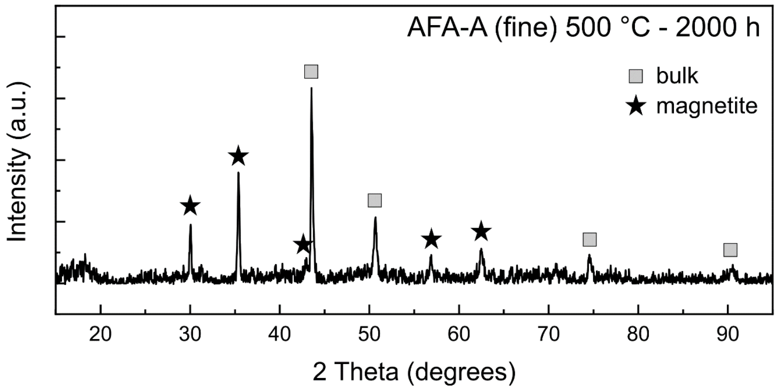

Figure S4: XRD pattern of fine ground surface of AFA-A after 2000 h exposure to liquid Pb at 500 °C.

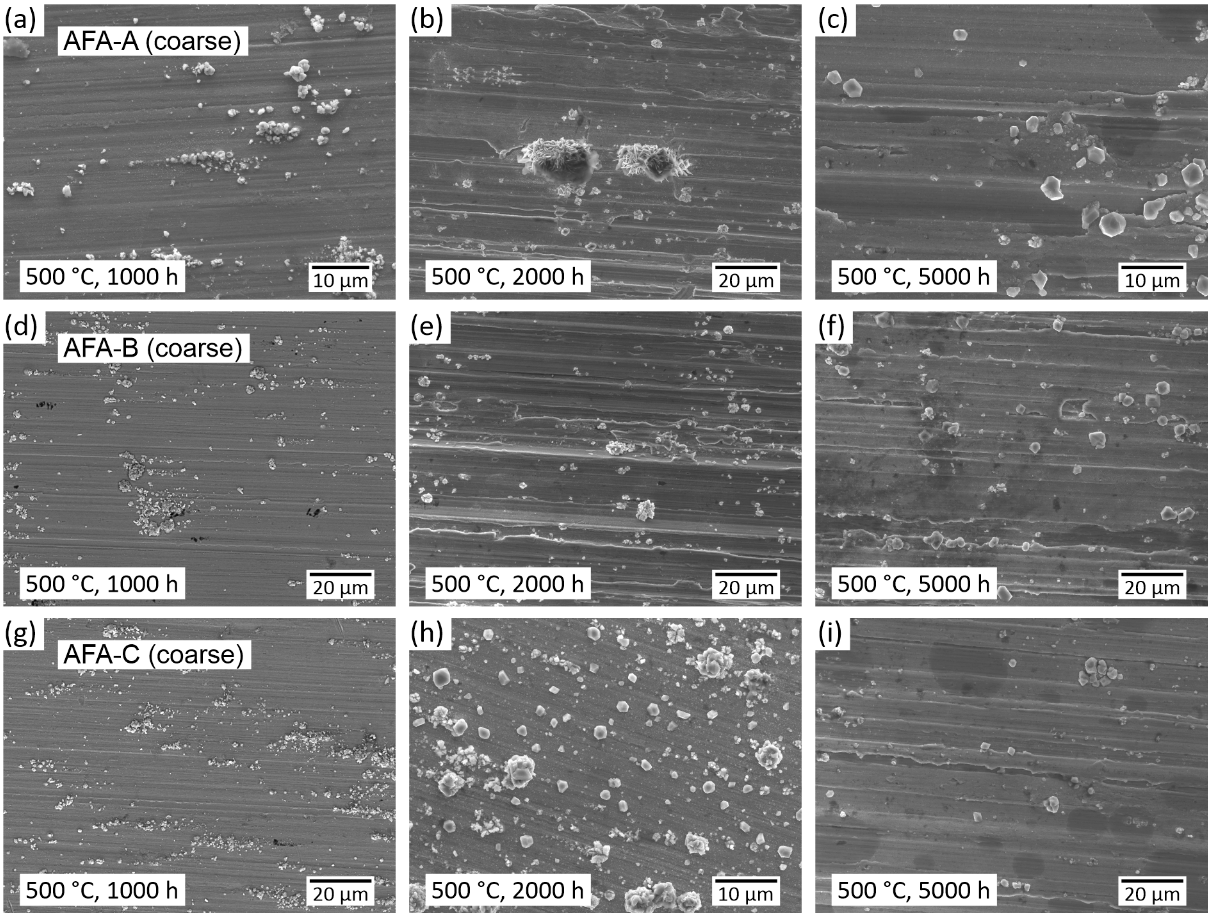

Figure S5: Coarse ground surfaces of AFA-A (a-c), AFA-B (d-f), and AFA-C (g-i) after exposure to liquid Pb at 500 °C, imaged in surface view by SEM-SE.

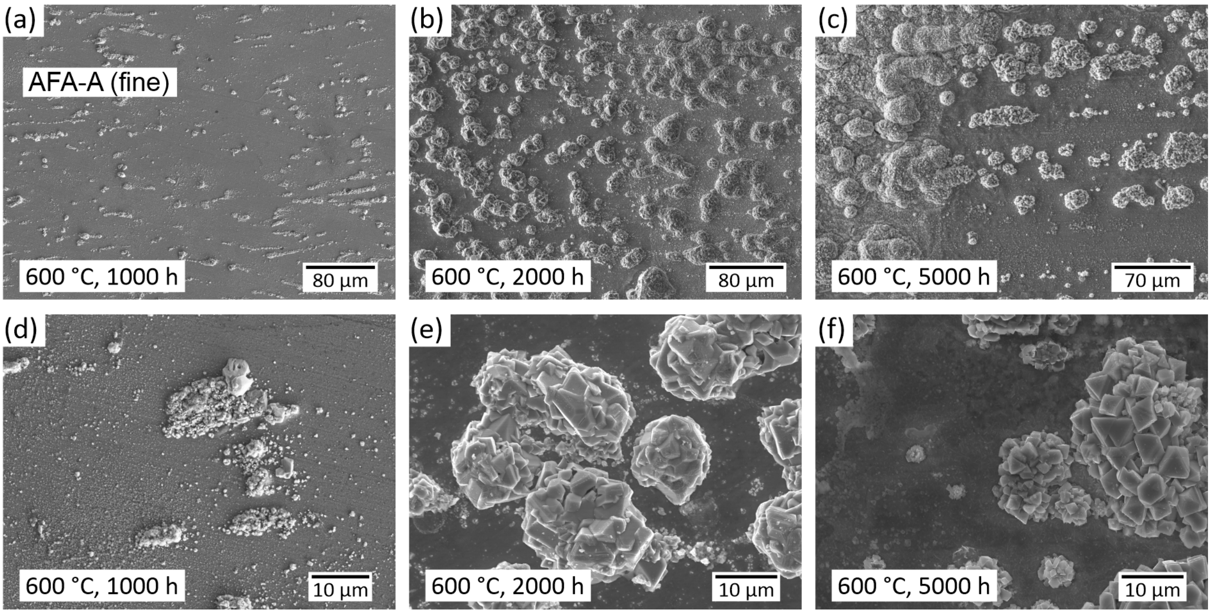

Figure S6: Fine ground surface of AFA-A after exposure to liquid Pb at 600 °C, imaged in surface view by SEM-SEM.

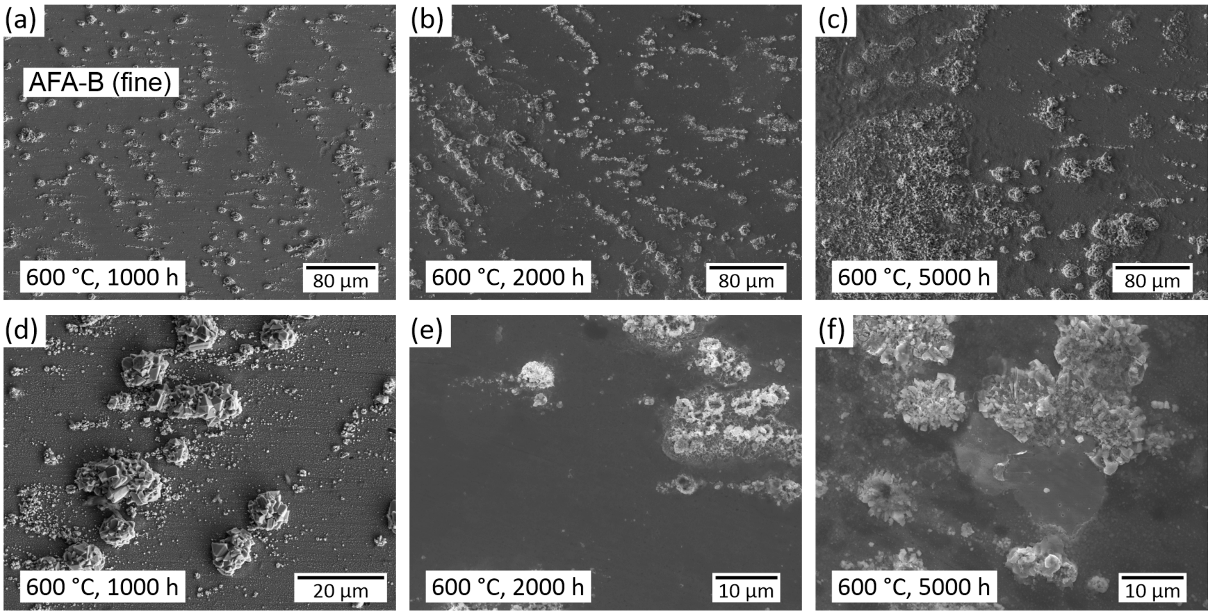

Figure S7: Fine ground surface of AFA-B after exposure to liquid Pb at 600 °C, imaged in surface view by SEM-SE.

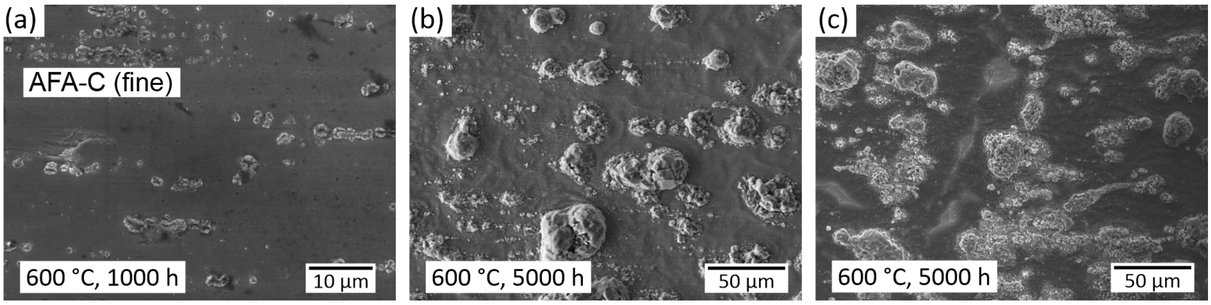

Figure S8: Fine ground surface of AFA-C after exposure to liquid Pb at 600 °C, imaged in surface view by SEM-SE.

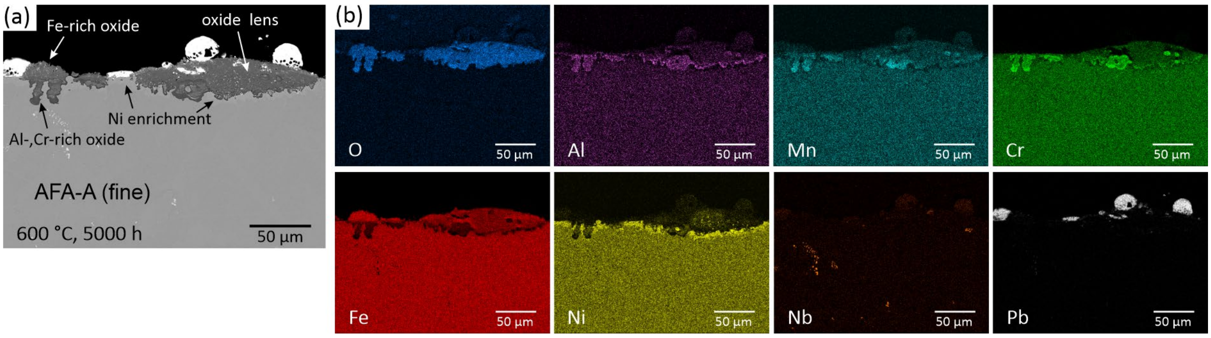

Figure S9: SEM-BSE cross-section image of fine ground surface of AFA-A after 5000 h exposure to liquid Pb at 600 °C (a) with corresponding EDX elemental mapping (b).

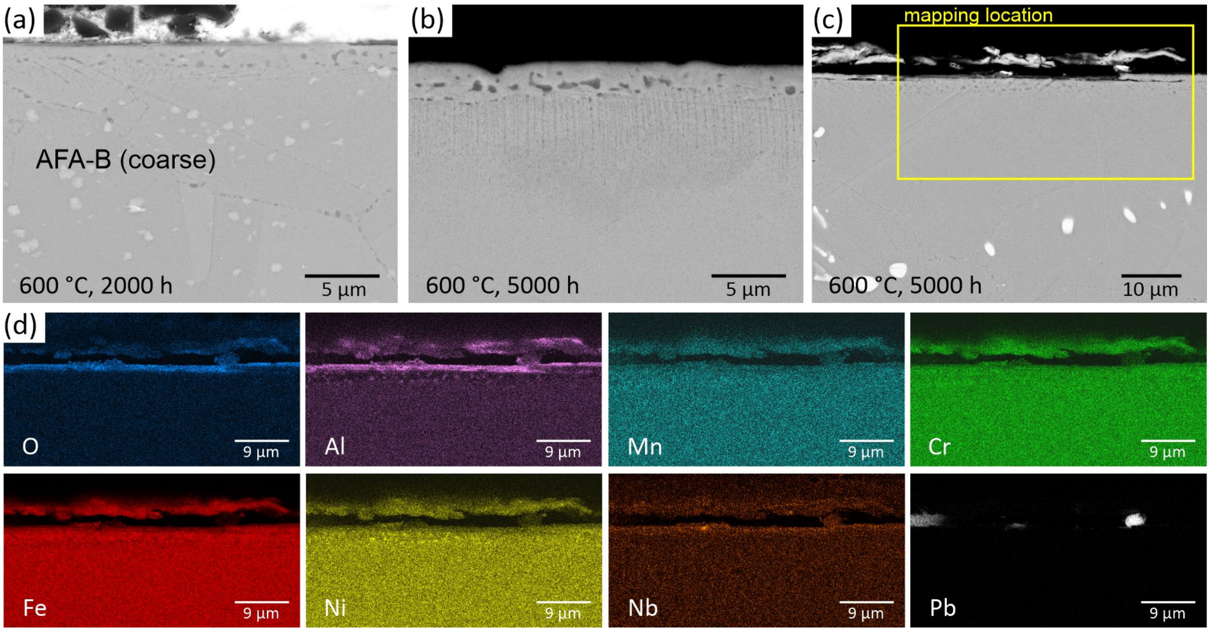

Figure S10: Cross-section analysis of coarse ground surface of AFA-B after exposure to liquid Pb at 600 °C. SEM-BSE images after 2000 h (a) and 5000 h exposure (b, c) with EDX elemental mapping of location shown in (c) presented in (d).
